# MR1-restricted T cell clonotypes are associated with “resistance” to *Mycobacterium tuberculosis* infection

**DOI:** 10.1172/jci.insight.166505

**Published:** 2024-05-08

**Authors:** Deborah L. Cross, Erik D. Layton, Krystle K.Q. Yu, Malisa T. Smith, Melissa S. Aguilar, Shamin Li, Elise C. Wilcox, Aude G. Chapuis, Harriet Mayanja-Kizza, Catherine M. Stein, W. Henry Boom, Thomas R. Hawn, Philip Bradley, Evan W. Newell, Chetan Seshadri

**Affiliations:** 1 Department of Medicine, University of Washington School of Medicine, Seattle, Washington, USA.; 2 Vaccine and Infectious Disease Division and; 3 Translational Science and Therapeutics Division, Fred Hutchinson Cancer Center, Seattle, Washington, USA.; 4 Department of Medicine, Makerere University, Kampala, Uganda.; 5 Department of Medicine and; 6 Department of Population and Quantitative Health Sciences, Case Western Reserve University, Cleveland, Ohio, USA.; 7 Public Health Sciences Division, Fred Hutchinson Cancer Center, Seattle, Washington, USA.

**Keywords:** Immunology, Infectious disease, T cell receptor, T cells

## Abstract

T cells are required for protective immunity against *Mycobacterium tuberculosis*. We recently described a cohort of Ugandan household contacts of tuberculosis cases who appear to “resist” *M*. *tuberculosis* infection (resisters; RSTRs) and showed that these individuals harbor IFN-γ–independent T cell responses to *M*. *tuberculosis*–specific peptide antigens. However, T cells also recognize nonprotein antigens via antigen-presenting systems that are independent of genetic background, known as donor-unrestricted T cells (DURTs). We used tetramer staining and flow cytometry to characterize the association between DURTs and “resistance” to *M*. *tuberculosis* infection. Peripheral blood frequencies of most DURT subsets were comparable between RSTRs and latently infected controls (LTBIs). However, we observed a 1.65-fold increase in frequency of MR1-restricted T (MR1T) cells among RSTRs in comparison with LTBIs. Single-cell RNA sequencing of 18,251 MR1T cells sorted from 8 donors revealed 5,150 clonotypes that expressed a common transcriptional program, the majority of which were private. Sequencing of the T cell receptor α/T cell receptor δ (TCRα/δ) repertoire revealed several DURT clonotypes were expanded among RSTRs, including 2 MR1T clonotypes that recognized mycobacteria-infected cells in a TCR-dependent manner. Overall, our data reveal unexpected donor-specific diversity in the TCR repertoire of human MR1T cells as well as associations between mycobacteria-reactive MR1T clonotypes and resistance to *M*. *tuberculosis* infection.

## Abstract 2

Mycobacteria-reactive MR1 clonotypes associate with resistance to <em>Mycobacterium tuberculosis</em> infection in human cohort.

## Introduction

Mycobacterium tuberculosis (M. *tuberculosis*) is the etiological cause of tuberculosis (TB), which caused an estimated 1.5 million deaths worldwide in 2020 (1). Nearly 1.7 billion people who have been exposed to *M*. *tuberculosis* are clinically asymptomatic but may harbor a latent TB infection (LTBI) on the basis of a positive tuberculin skin test (TST) or IFN-γ release assay (IGRA) (2, 3). Most animal models seek to recapitulate active disease rather than latent *M*. *tuberculosis* infection, so the immune mechanisms underlying protection from *M*. *tuberculosis* infection rather than disease are currently undefined (4). We recently described a cohort of Ugandan household contacts of active TB cases who do not convert their TST or IGRA despite high risk of exposure to *M*. *tuberculosis* (5, 6). We further showed that these individuals have primed IFN-γ–independent T cell responses to *M*. *tuberculosis*–specific antigens, confirming the epidemiologic evidence of exposure (7). Since these individuals also did not develop active TB over a median of 9.5 years of follow-up, we hypothesize that they “resist” *M*. *tuberculosis* infection (and refer to them as resisters; RSTRs). Whether other T cell profiles are also associated with the RSTR phenotype is currently unknown.

T cells typically recognize foreign peptide antigens through a genetically rearranged T cell receptor (TCR) when bound to highly polymorphic major histocompatibility complex (MHC) molecules (8). T cells are also capable of recognizing nonpeptide antigens through MHC-independent antigen presentation systems (9). For example, T cells are activated by lipids and small molecules presented by cluster of differentiation 1 (CD1) and MHC-related protein 1 (MR1), respectively (10, 11). Further, T cells with a γδ TCR can recognize nonpeptide antigens presented by butyrophilin molecules, CD1, and MR1 (12–14). Because CD1, MR1, and butyrophilin exhibit limited sequence diversity between donors, the T cells that act through these systems are called donor-unrestricted T cells (DURTs) (15).

MR1-restricted T cells are strongly activated by derivatives of the riboflavin biosynthesis pathway and capable of lysing bacterially infected cells (11, 16). Mucosal associated invariant T (MAIT) cells are a subset of MR1T cells that are abundant in human peripheral blood and characterized by expression of a germline-encoded TCRα chain utilizing TRAV1-2 and TRAJ33, TRAJ20, or TRAJ12 gene segments and staining with MR1-5-(2-oxopropylideneamino)-6-d-ribitylaminouracil–loaded (MR1-5-OP-RU–loaded) tetramer (10, 17, 18). X-ray crystallography studies have demonstrated the importance of the TCRα chain in recognition of MR1-presented antigens, with additional reports demonstrating a role for TRBV genes in differential bacterial sensing (11, 19–22). In addition, there is a growing appreciation of MR1T cells that lack the conserved TCR gene usage that characterizes MAIT cells and may mediate recognition of alternative ligands (23). For example, photolumazines were recently identified as a novel class of mycobacterium-derived ligands that could be presented by MR1 to MAIT cells (24).

Here, we sought to comprehensively study the association between DURTs and “resistance” to *M*. *tuberculosis* infection. We found that the frequency of circulating MR1T cells was increased in RSTRs when compared with matched LTBI controls. This result led to a detailed study of MR1T cells using multimodal single-cell RNA-Seq (scRNA-Seq). Surprisingly, we found that over 80% of MR1T clonotypes were not shared among multiple donors, highlighting an unexpected donor specificity of this DURT subset. At least 2 of these MR1T clonotypes were preferentially expanded in the blood of RSTRs and were activated by mycobacteria-infected cells in a TCR-dependent manner. A broader survey of TCRα clonotypes defined by immunosequencing also revealed preferential clone sharing among RSTRs when compared with LTBI controls. Together, these data reveal associations between MR1-restricted as well as other DURT clonotypes of unknown specificity and “resistance” to *M*. *tuberculosis* infection.

## Results

### MR1T cells are expanded in the peripheral blood of RSTRs compared with LTBI controls.

**Figure 1:**
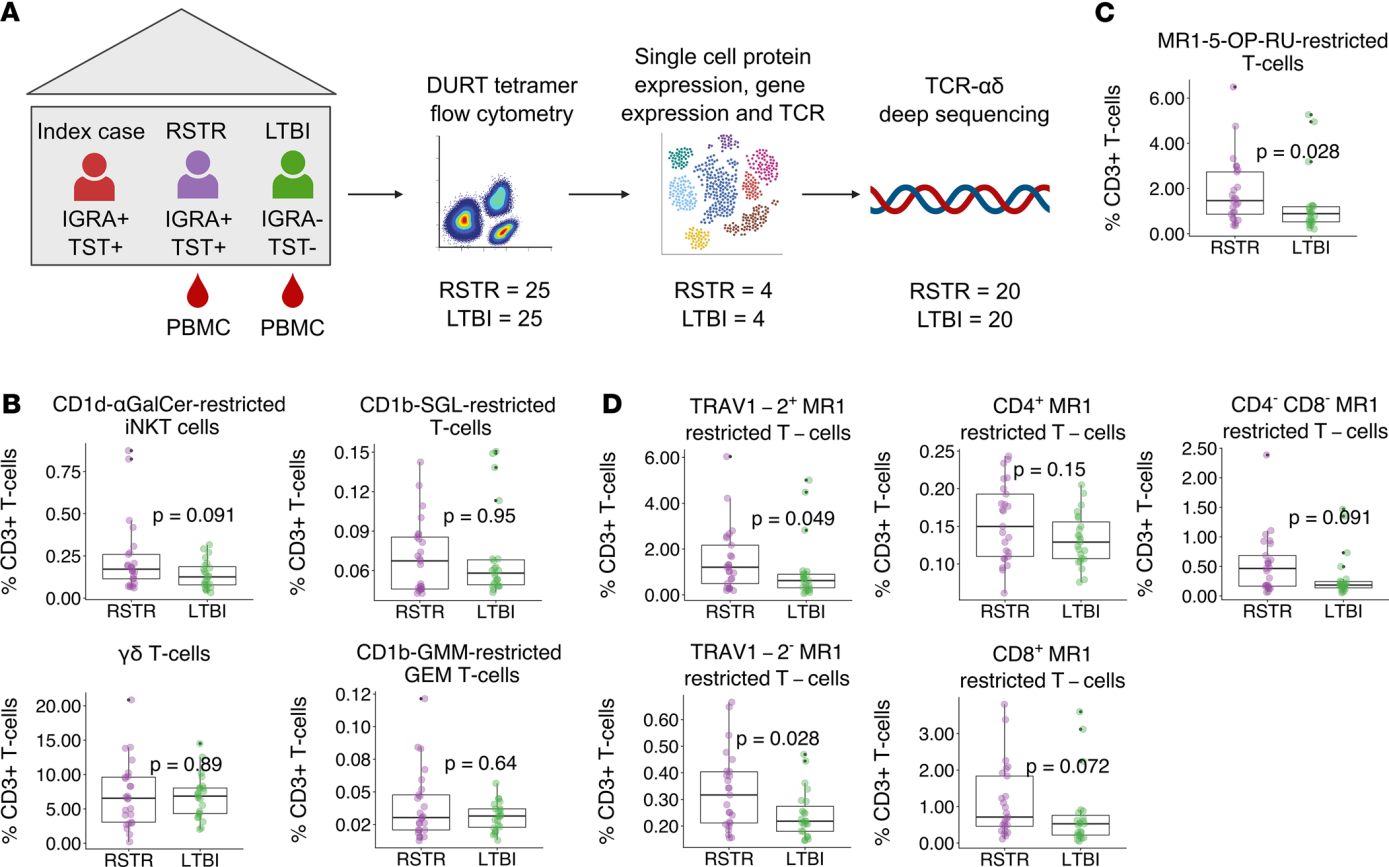
MR1T cells are expanded in the peripheral blood of RSTRs compared with LTBI controls. (**A**) Overview of study design. Household contacts of active TB cases were classified as RSTR or LTBI as defined by longitudinal testing with TST and IGRA. Combinatorial tetramer staining and multiparameter flow cytometry were used to quantify frequencies of DURTs in RSTRs (*n* = 25) and LTBI cases (*n* = 25). MR1-5-OP-RU tetramer was used to sort MR1T cells for multimodal scRNA-Seq from RSTRs (*n* = 4) and LTBI cases (*n* = 4), including identification of TCR clonotype, transcriptional profiling, and surface protein expression with Cellular Indexing of Transcriptomes and Epitopes sequencing (CITE-Seq). Deep sequencing of the TCRα and TCRδ repertoire was performed using the immunoSEQ platform (Adaptive Biotechnologies) on RSTRs (*n* = 20) and LTBIs (*n* = 20). (**B**) Frequencies of CD1-restricted and γδ T cells stratified by group as a proportion of live CD3^+^ T cells. (**C**) Frequencies of MR1-5-OP-RU–staining T cells as a proportion of live CD3^+^ T cells stratified by group. (**D**) Among MR1–OP-RU–staining T cells, the frequencies of T cells expressing CD4, CD8, or TRAV1-2 as a proportion of live CD3^+^ T cells stratified by group. Statistical testing was performed using the Wilcoxon rank sum test and unadjusted *P* values are displayed. A *P* value of less than 0.05 was considered significant. For all box plots, the upper whisker extends to the highest value within 1.5× IQR, and the lower whisker extends to the lowest value within 1.5× IQR. The horizontal line in the center of the box reflects the median value of the data. α-GalCer, α-galactosylceramide.

We leveraged samples collected as part of a longitudinal study of TB household contacts in Uganda, as we have previously reported (5, 6). Three sequential IGRAs, measured by QuantiFERON-TB Gold, were performed on blood samples, and 1 additional TST was performed as part of this retracing study that took place approximately 9.5 years after the initial *M*. *tuberculosis* exposure (6). Human immunodeficiency virus–negative (HIV^–^) individuals who remained concordantly negative for all tests were defined as RSTRs, and control individuals with LTBI were defined by consistently positive results at all time points by both IGRA and TST. For this analysis, we selected a representative subset of RSTRs and LTBI controls after matching by age (≥15 years), sex, and epidemiologic risk score (Supplemental Table 1; supplemental material available online with this article; https://doi.org/10.1172/jci.insight.166505DS1). We used combinatorial tetramer staining and multiparameter flow cytometry to quantify the frequencies of DURTs in RSTRs (*n* = 25) and LTBI cases (*n* = 25) (25) (Figure 1A and Supplemental Figure 1B). We observed no difference in circulating frequencies of γδ T cells, invariant natural killer T cells, glucose monomycolate–specific (GMM-specific), or diacylated sulfoglycolipid–specific (Ac_2_SGL-specific) T cells between RSTRs and LTBI controls (Figure 1B). However, circulating frequencies of MR1T cells were 1.65-fold higher in RSTRs when compared with LTBI controls (Figure 1C, *P* = 0.028). As a proportion of live cells, we observed a similar trend, though this did not reach significance (*P* = 0.056, data not shown). Frequencies of subsets defined by TRAV1-2 or coreceptor (CD4 or CD8) expression all showed a similar trend toward expansion in RSTRs (Figure 1D). Taken together, these data highlight the association between circulating frequencies of MR1T cells and “resistance” to *M*. *tuberculosis* infection.

### The majority of the MR1-restricted TCR repertoire consists of donor-specific clonotypes.

**Figure 2:**
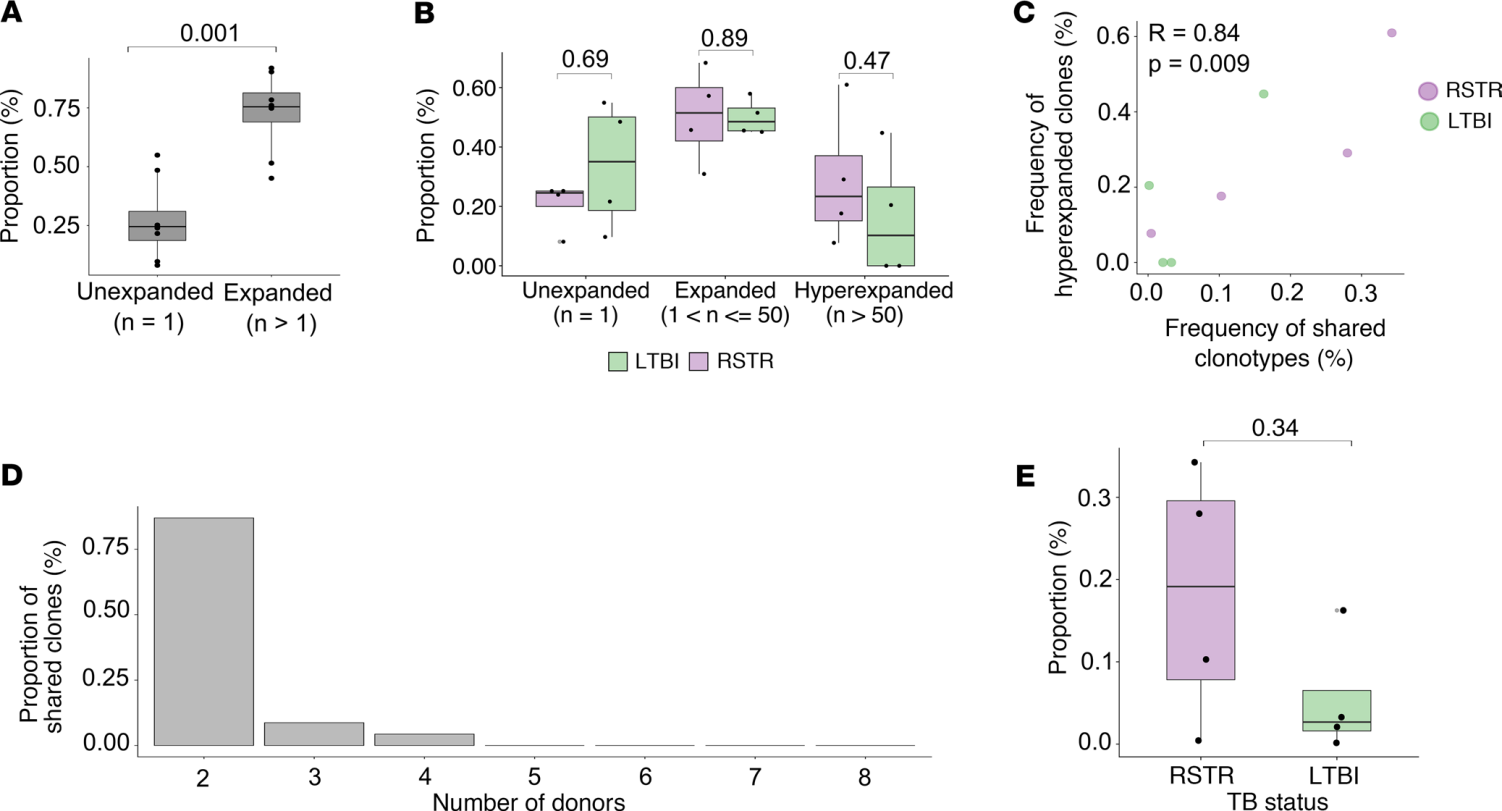
The majority of the MR1-restricted TCR repertoire consists of donor-specific clonotypes. (**A**) The proportion of unexpanded (*n* = 1) and expanded (*n* > 1) MR1T clonotypes as defined by nucleotide sequence is displayed as a percentage of each donor’s total repertoire (*n* = 8). (**B**) The proportion of unexpanded (*n* = 1), expanded (1 < *n* ≤ 50), and hyperexpanded (*n* > 50) MR1T clonotypes as a percentage of the total repertoire is displayed stratified by group. Height of the bar reflects the mean, and error bars represent standard deviation. (**C**) Pearson correlation between the frequency of hyperexpanded clonotypes and frequency of public clonotypes. Each point represents 1 donor, and points are colored according to group. (**D**) Among shared clones, the proportion detected in 2 or more donors is shown. (**E**) The proportion of shared MR1T clonotypes is displayed stratified by group. Each point is an individual donor. Statistical testing was performed using the Wilcoxon rank sums test and unadjusted *P* values are displayed. A *P* value of less than 0.05 was considered significant. For all box plots, the upper whisker extends to the highest value within 1.5× IQR, and the lower whisker extends to the lowest value within 1.5× IQR. The horizontal line in the center of the box reflects the median value of the data.

Clonotypic diversity in the MR1T cell compartment is generally thought to be low given the majority of clonotypes express a semi-invariant TCR (23). However, evidence of MR1T cell populations with greater TCR diversity has been reported (26). Therefore, to determine whether MR1T cell clonotypic diversity is associated with *M*. *tuberculosis* “resistance,” we sorted MR1-5-OP-RU^+^ T cells from RSTRs (*n* = 4) and LTBI cases (*n* = 4) and performed multimodal scRNA-Seq on 70,535 MR1T cells. Following pre-processing, 18,251 cells with paired scTCR-Seq information were included in downstream analysis (Supplemental Figure 2A). Across all donors (*n* = 8), 5,150 TCR clonotypes were identified, defined by both complementarity determining region 3α (CDR3α) and CDR3β nucleotide sequences. The number of unique clonotypes, overall richness, and repertoire clonality were similar between RSTRs and LTBI cases (Supplemental Figure 2B). Consistent with the prior literature, we found that the majority of the MR1T repertoire was composed of expanded clonotypes that appeared at least twice in our data set (27). On average, expanded clonotypes represented 75% of the MR1T repertoire (Figure 2A). Surprisingly, we also observed a large minor fraction (~25%) of each donor’s repertoire that was unexpanded, appearing only once in the repertoire. The proportions of unexpanded (*n* = 1), expanded (1 < *n* ≤ 50), and hyperexpanded (*n* > 50) MR1T cell clonotypes were highly variable among donors (Supplemental Figure 2C), but few differences in proportions were noted between RSTR and LTBI (Figure 2B).

Given the extent of clonal expansion and the designation of MR1T cells as DURTs, we hypothesized that the majority of expanded clonotypes might be shared between donors. Frequencies of shared clonotypes were strongly correlated with the frequency of hyperexpanded clonotypes (*R* = 0.84, *P* = 0.010), suggesting shared clonotypes represented a major fraction of the total repertoire (Figure 2C). To our surprise, only 23 of 5,150 unique clonotypes in our data set were shared among 2 or more donors, with 20 (87%) of 23 clonotypes being shared only between 2 individuals (Figure 2D). Finally, we found that the frequency of shared clonotypes trended higher in RSTRs compared with LTBI donors (*P* = 0.34) (Figure 2E). Taken together, these data reveal higher-than-expected donor restriction in the MR1T repertoire, with only a minor proportion of highly expanded clonotypes being shared between donors.

### MR1T clonotype diversity is constrained by TCR gene usage and CDR3 length.

**Figure 3:**
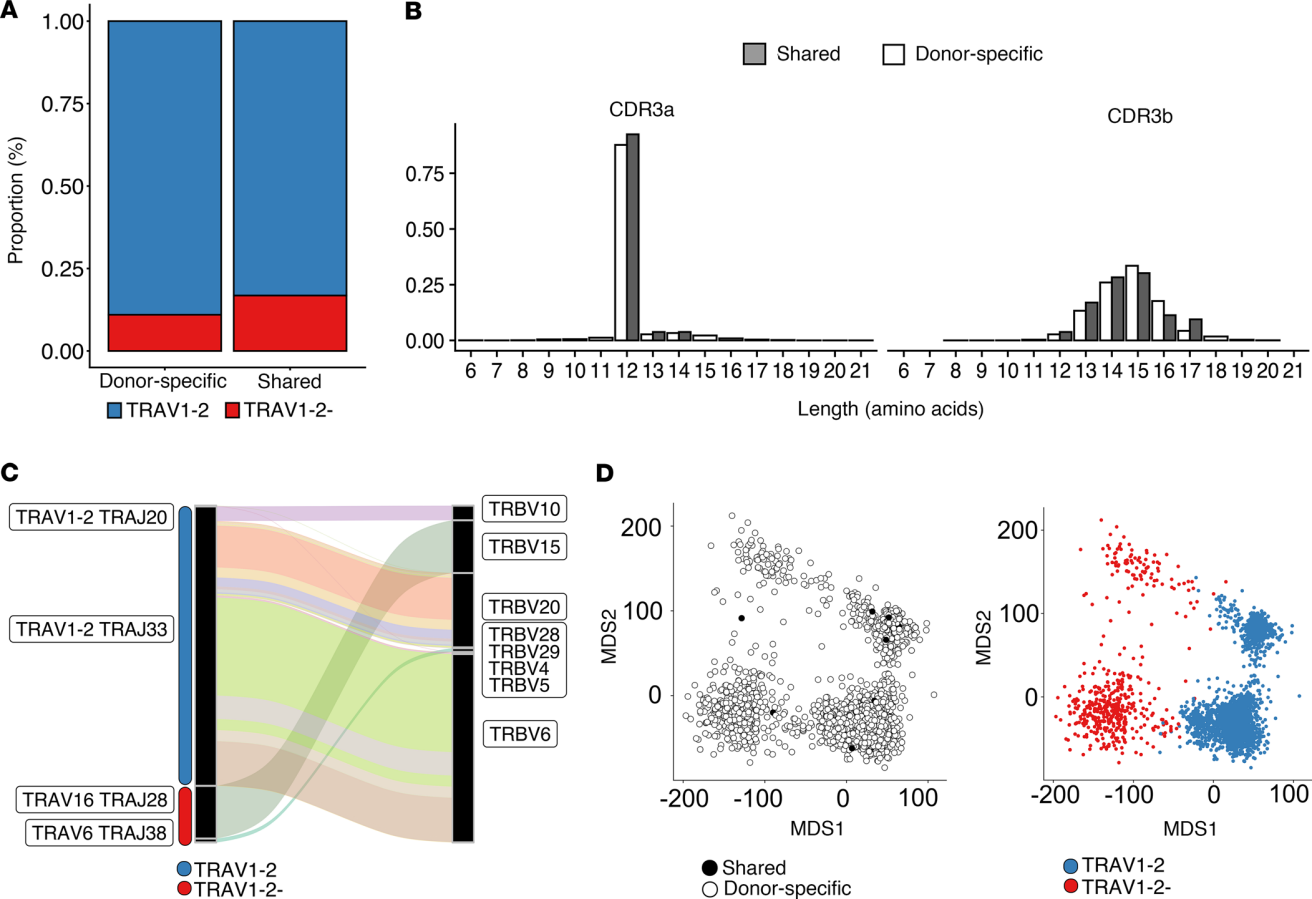
MR1T clonotype diversity is constrained by TCR gene usage and CDR3 length. (**A**) Bar plot showing the median proportion of TRAV1-2^+^ and TRAV1-2^–^ MR1T cells per donor (*n* = 8). (**B**) Histogram of proportion of CDR3α and CDR3β amino acid sequence lengths for shared and donor-specific clonotypes. Shaded = shared, white = donor specific. (**C**) Sankey diagram showing the gene usage of shared MR1T clonotypes (*n* = 23 clonotypes). A clonotype was defined by nucleotide sequence and considered shared if it was identified in 2 or more donors. TRAV-TRAJ gene rearrangement and TRBV gene usage are labeled. The width of each connection in the plot represents the proportion of that clonotype within the shared repertoire. (**D**) Multidimensional scaling (MDS) plot of the TCRdist distance matrix representing the relative similarity among CDR3 sequences in 2-dimensional space (*n* = 5,150 clonotypes). Each dot represents 1 sequence and is colored by TRAV1-2 gene usage and whether it is donor specific or shared.

We next sought to gain further insight into the features that distinguish shared from donor-specific MR1T clonotypes. In both compartments, the majority of clonotypes expressed TRAV1-2, consistent with published data and validating our sorting strategy (19) (Figure 3A). In line with this observation, we identified high proportions of donor-specific and shared clonotypes with a fixed CDR3α sequence length of 12 amino acids, consistent with known molecular requirements for binding of the MR1-5-OP-RU complex (15, 28) (Figure 3B). In addition to TRAV1-2^+^ clones, 2 TRAV1-2^–^ clonotypes were identified as being shared between donors (Figure 3C). This included a TRAV16-TRAJ28 clonotype paired with TRBV15 and a TRAV6-TRAJ38 clonotype paired with TRBV29-1. Both TRAV1-2^–^ clonotypes had CDR3α sequences that lacked the TRAJ-associated conserved tyrosine at position 95, which has been previously identified as critical for recognition of MR1 by TRAV1-2^+^ clonotypes, and had variable CDR3α lengths.

To evaluate the similarity between CDR3 sequences in the donor-specific and shared repertoires, we clustered TCR clonotypes based on both CDR3α and CDR3β amino acid sequences using TCRdist (29). Multidimensional scaling revealed differences in CDR3 similarity that were influenced by TRAV1-2 expression (Figure 3D). We noted that shared and donor-specific clonotypes did not cluster independently in this analysis, suggesting a high degree of sequence concordance between shared and donor-specific compartments (Figure 3D). Furthermore, we identified TRAV1-2^+^ MAIT-like TCRs that are similarly donor restricted yet appear to have high CDR3 sequence homology with canonical MAIT TCRs. Taken together, these data identify a broad array of non–TRAV1-2 MR1-restricted TCRs that are reactive with 5-OP-RU and are typically donor restricted.

### MR1T cells are characterized by transcriptional homogeneity at rest.

**Figure 4:**
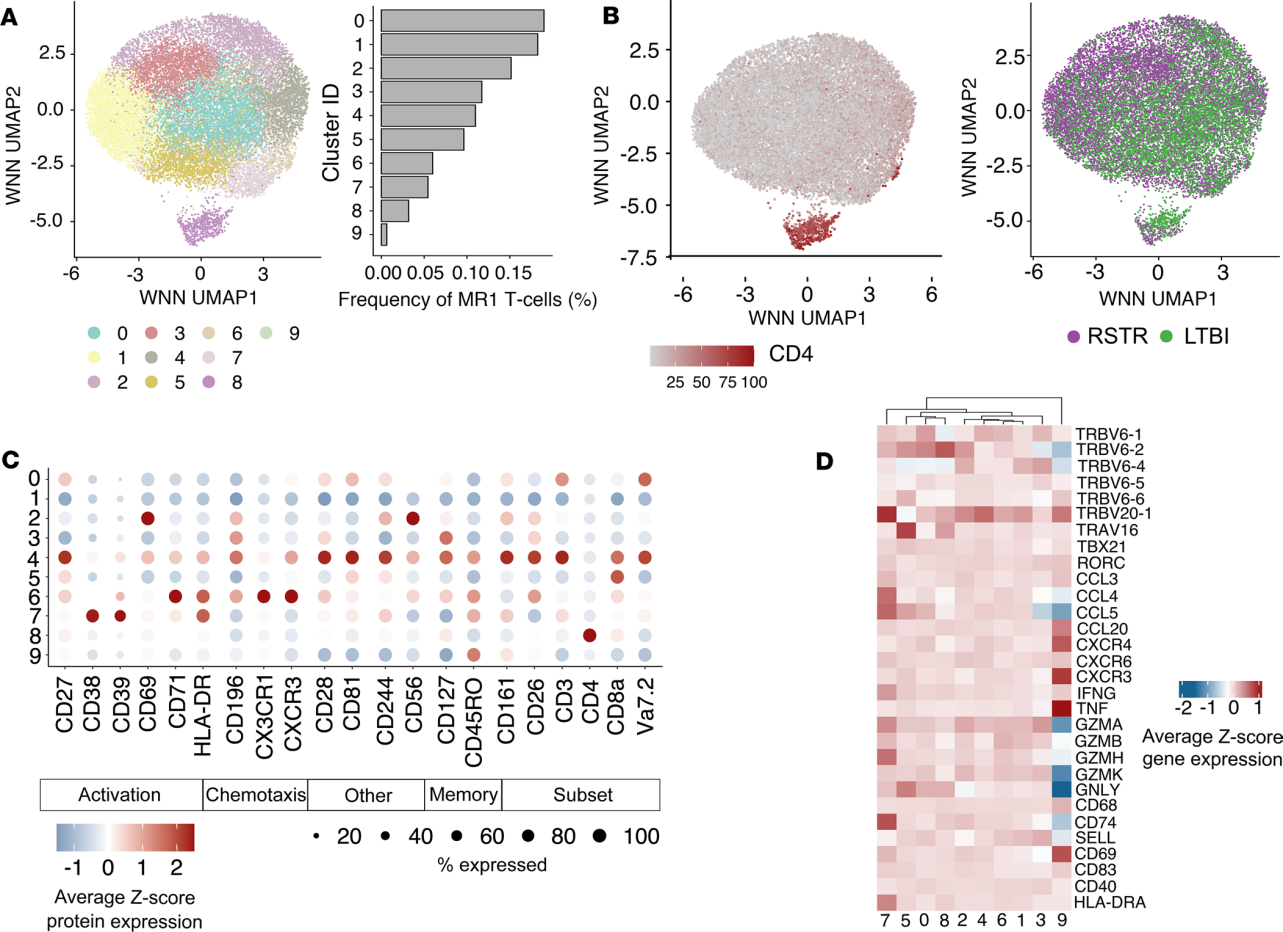
MR1T cells are characterized by transcriptional homogeneity at rest. (**A**) Weighted nearest neighbor (WNN) analysis was used to cluster transcriptional profiles and cell surface protein expression of 18,251 sorted MR1-5-OP-RU^+^ MR1T cells that could be annotated with both a TCRα and TCRβ sequence. Uniform manifold approximation and projection (UMAP) was used to visualize the results. The number of cell barcodes constituting each cluster is displayed in the bar chart. (**B**) UMAP representation of MR1T cells colored by either relative expression of CD4 surface protein or TB group. (**C**) Dot plot showing the scaled, normalized expression of cell surface protein expression across all WNN-identified clusters. Dot size reflects the proportion of cells in a given cluster that express a marker. Dots are colored by average *z* score protein expression. (**D**) Heatmap of normalized, transformed gene expression of selected genes known to be associated with MR1T cell function.

To gain further insight into the phenotypic diversity of MR1T cells, we simultaneously analyzed single-cell protein and gene expression data on sorted MR1T cells (30) (Figure 4A). After quality control filtering, a total of 18,251 cells with paired TCRα and TCRβ chains were analyzed with an average of 1,876 cells per donor, which did not differ between groups (Supplemental Figure 2D) (*P* = 0.89). WNN analysis of protein and gene expression data revealed 10 clusters (Figure 4A and Supplemental Table 2). Surface protein expression was a major driver of cell clustering, in particular, expression of CD4 (Figure 4B). All donors contributed to all clusters in our analysis (data not shown). Further, frequencies of WNN-defined clusters did not appear to be associated with RSTR status (Figure 4B). Genes associated with known MR1T cell functions, including IFN-γ, cytotoxicity, and activation, had similar expression across all clusters, suggesting limited functional delineation between clusters (Figure 4D). In keeping with this observation, transcription factors known to define MR1T cell functional subsets in mice, RORγ and T-bet, were not significantly differentially expressed between clusters in our data (31, 32) (Figure 4D). Surface protein expression revealed subtle differences in the magnitudes of expression of activation and memory, but the proportion of cells in each cluster expressing a particular protein was broadly similar (Figure 4C). Analysis of covariation between gene expression and TCR sequence using the clonotype neighbor graph analysis (CoNGA) package highlighted 1 small cluster of CD4-expressing T cells with diverse TCR sequences as having significant gene expression/TCR correlation (Supplemental Figure 3) (33). No other significant clusters were identified, consistent with overall transcriptional homogeneity. Taken together, these data reveal that MR1T cells exhibit remarkably homogeneous transcriptional programs at rest despite clonotypic diversity.

### MR1T cell and DURT clonotypes are enriched among RSTRs.

To determine the prevalence of identified MR1T clonotypes in a larger number of donors and robustly examine their association with resistance, we performed deep sequencing of the TCRα and TCRδ chains present in the peripheral blood of RSTRs (*n* = 19) and LTBI controls (*n* = 20).

**Figure 5:**
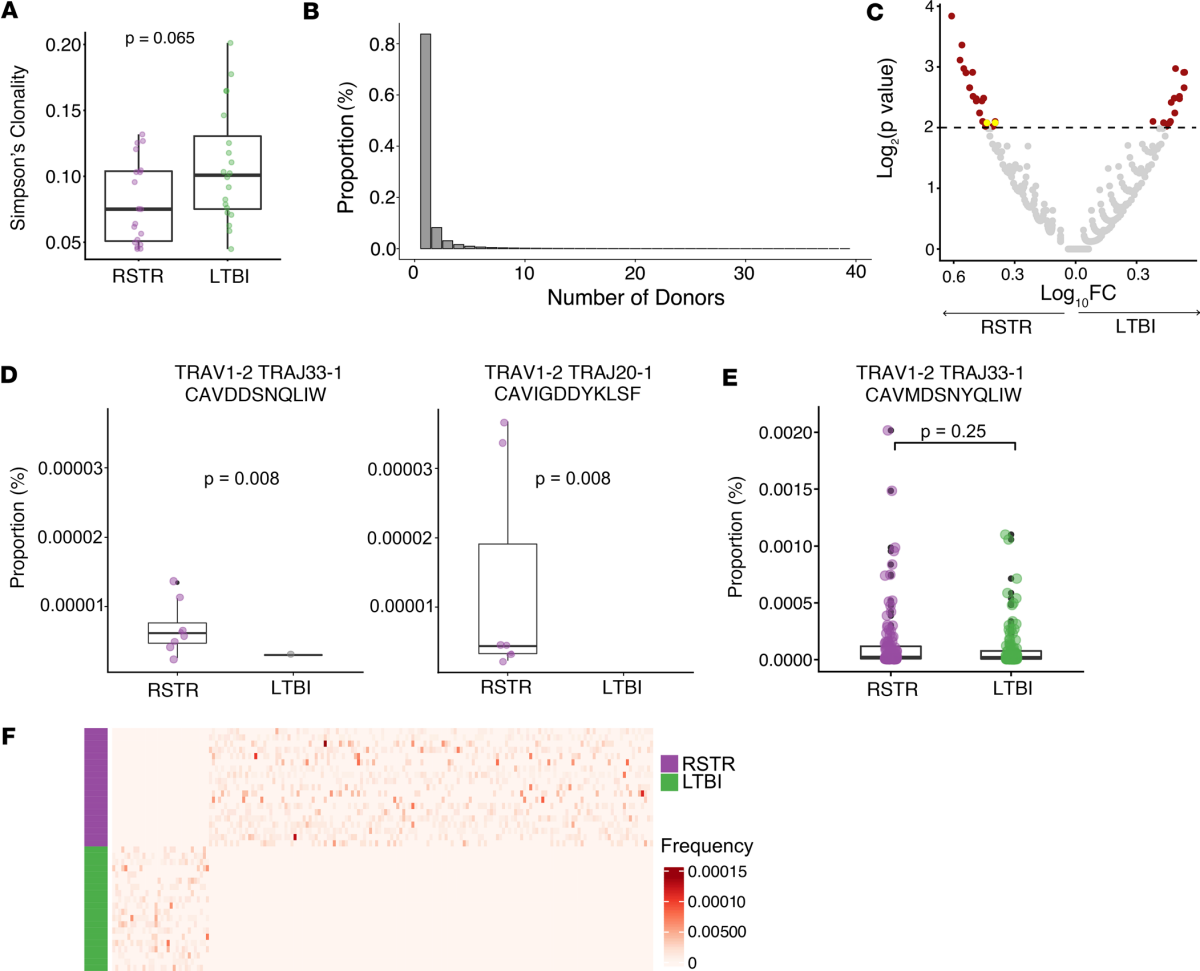
Immunosequencing reveals MR1T and DURT clonotypes that are significantly enriched among RSTRs. (**A**) PBMCs from RSTR (*n* = 19) and LTBI (*n* = 20) were analyzed using TCRα/δ immunoSEQ (Adaptive Biotechnologies). Simpson’s clonality was calculated for each donor’s repertoire and stratified by group. (**B**) Bar plot showing the number of TCRα/δ clonotypes that were shared between donors (*n* = 39) (**C**) Volcano plot of TCRα/δ clonotypes after testing for enrichment in RSTR or LTBI groups. Enriched clones are highlighted in red. MR1T clonotypes identified previously in scTCR-Seq are highlighted in yellow. A clonotype was defined by CDR3α amino acid sequence and V- and J-gene identity. Enriched clones were identified by 2-sided Fisher’s exact test using a *P* value threshold of 0.01 (adjusted for multiple testing) (threshold indicated by dashed line on the plot). (**D**) Box plot of frequencies of MR1T clonotypes previously defined in scTCR-Seq analysis that were also identified among enriched clones in the volcano plot. Matching was based on CDR3α amino acid sequence and V- and J-gene identity. Displayed *P* values were calculated using Fisher’s exact tests and are unadjusted for multiple testing. (**E**) Box plot of frequency of canonical MAIT TCRα stratified by group. Displayed *P* value was calculated using Wilcoxon rank sum test. (**F**) Heatmap of frequencies (as a proportion of templates per sample) of TCRα/δ clonotypes found exclusively among either RSTR or LTBI donors and *P* < 0.05 by Fisher’s exact test. Unadjusted *P* values were used to select clonotypes for inclusion in the heatmap. A *P* value of less than 0.05 was considered significant. For all box plots, the upper whisker extends to the highest value within 1.5× IQR, and the lower whisker extends to the lowest value within 1.5× IQR. The horizontal line in the center of the box reflects the median value of the data.

We identified a median of 80,227 and 74,915 templates per donor in RSTRs and LTBI cases, respectively (*P* = 0.69). There was a trend toward decreased clonality among RSTRs (*P* = 0.065) (Figure 5A). We identified 306,482 clonotypes that were shared between at least 2 donors, representing 16.4% of the total repertoire. Notably, the distribution of TCRα clone sharing in this cohort mirrored what we observed in the scTCR-Seq data, with 8.01% of templates being shared between 3 or more donors (Figure 5B). Using 2-sided Fisher’s exact tests and a nominal *P* value cutoff of 0.01, we identified 303 clonotypes that were significantly associated with either RSTR or LTBI status (Figure 5C). Of these enriched clonotypes, 2 clonotypes were identical to TCRα sequences present in sorted MR1T cells, and both were preferentially detected among RSTRs (Figure 5D). Notably, the canonical MAIT TCRα was detected at similarly high frequency among both RSTRs and LTBI cases (Figure 5E). None of the MR1T clonotypes from our scTCR-Seq analysis were found to be enriched among LTBI donors (data not shown). Beyond MR1T clonotypes, 301 additional TCRα or TCRδ chains of unknown restriction were significantly enriched in either the RSTR or LTBI controls. A larger number of clonotypes were exclusively shared among RSTRs (*n* = 147) versus LTBI donors (*n* = 33) (*P* < 0.0001, χ^2^ test) (Figure 5F). Together, these data reveal that MR1T cell and DURT clonotypes of unknown specificity are associated with resistance to *M*. *tuberculosis* infection.

### MR1T clonotypes associated with RSTRs recognize mycobacteria-infected cells.

**Figure 6:**
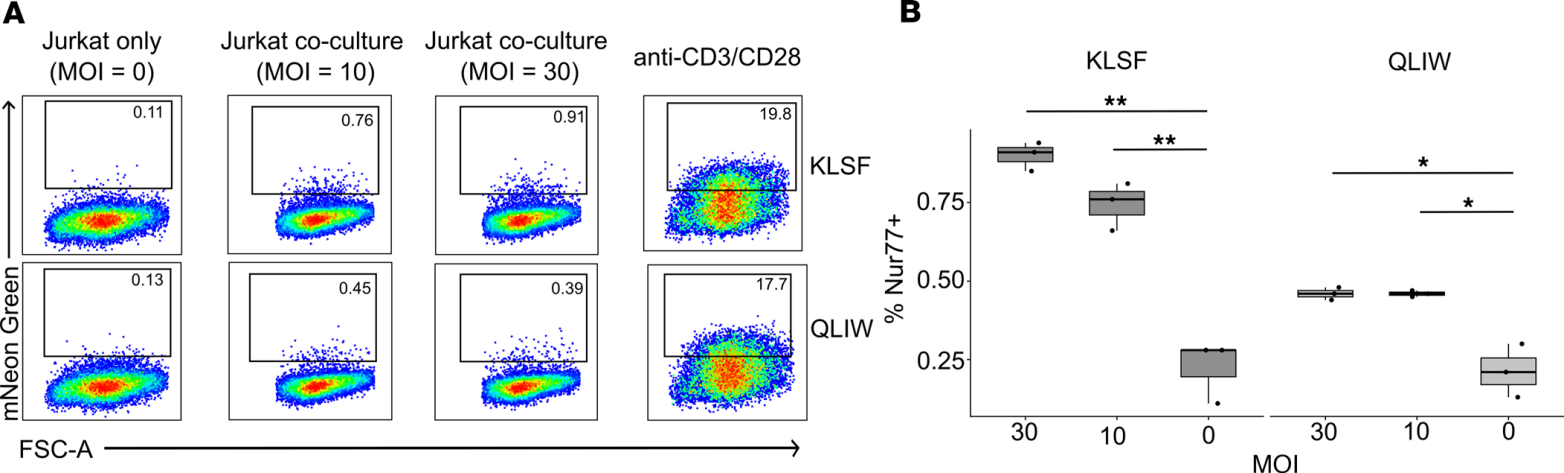
MR1T clonotypes associated with RSTRs recognize mycobacteria-infected cells. (**A**) Gating scheme identifying Nur77^+^ events in cultures stimulated with anti-CD3/CD28, BCG-infected A549 cells (MOI = 10, MOI = 30) or media only. (**B**) The proportion of Nur77^+^ events after coculture with infected A549 cells at an MOI of 30 and 10 (*n* = 3). Figure shows representative example of 3 experiments. *P < 0.05, **P < 0.01.

Finally, we sought to examine the functional importance of MR1T clonotypes preferentially enriched in RSTRs in our scTCR-Seq analysis and corroborated by TCRα/δ sequencing. We stably expressed enriched TCRs in Jurkat reporter cell lines and assessed for TCR-dependent recognition of A549 cells infected with live Bacillus Calmette-Guerin (BCG). The characteristics of selected MR1T clonotypes are summarized in Table 1. Nur77 expression, which is indicative of signaling through the TCR, was robustly detected after CD3/CD28 stimulation and absent in Jurkat cell lines cultured alone or cocultured with uninfected A549 cells (Figure 6A). Under experimental conditions, both MR1-restricted TCRs were found to recognize BCG-infected A549 cells in a dose-dependent manner (Figure 6, A and B). Taken together, these findings reveal that RSTR-associated MR1T clonotypes are activated by mycobacteria-infected antigen-presenting cells in a TCR-dependent manner.

## Discussion

“Resistance” to *M*. *tuberculosis* infection may be mediated through both innate and adaptive immune mechanisms that are not mutually exclusive (34). Since DURTs have features of both innate and adaptive immunity, we hypothesized that they might play a role in this unique clinical phenotype. In this study, we observed that RSTR individuals had significantly higher frequencies of peripheral blood MR1T cells in comparison with latently infected donors (Figure 1C). Previous studies have observed that MAIT cell frequencies in humans are highly variable between donors (35). The reason for this is likely to be multifactorial and involve genetic and developmental factors. Single-nucleotide polymorphisms modulating MAIT cell frequencies in peripheral blood have been characterized, suggesting that donor genetic background plays a key influence (36). Other factors, including early colonization of the intestinal tract with commensal bacteria after birth, are key events that drive MAIT cell expansion in mice (37). The lack of MAIT cells in germ-free animals indicates that the microbiome is a critical antigen source necessary for maintenance of circulating frequencies (38). In addition, diversity of the microbiome in humans has been found to correlate positively with maintenance of higher MAIT cell cytotoxic function and frequency (39). Overall, these reports indicate that donor genetics and microbiota composition are key factors in MR1T cell biology and may affect the phenotype we observe here.

In this study, we report a relatively diverse TCR repertoire among sorted MR1T cells. This finding was surprising as the majority of MR1T cells are expected to express a semi-invariant TCR (9). However, reports of diverse MR1T clonotypes expressing noncanonical receptors have been previously published, and it has been suggested that these TCRs could mediate recognition of a wider range of antigens. A T cell clone expressing a noncanonical TCR was shown to effectively kill cancer cells presenting endogenous ligands via MR1 (40). Furthermore, diverse MR1-restricted clonotypes have been shown to differentially recognize bacterial and fungal pathogens in vitro, and pathogen-specific expansion of MR1-restricted clonotypes has been reported in the context of acute *Salmonella* Paratyphi A infection (21, 22, 27, 28). At least one other study comparing TCR repertoires of MAIT cells in the blood and liver of 4 healthy donors using scTCR-Seq noted a pattern of diversity that was largely donor restricted, similar to our observations here (41). Though their transcriptional profiles appear relatively homogeneous, diverse TCRs may perform a biologically critical role in the recognition of diverse ligands.

We report an association between specific MR1T clonotypes and “resistance” to *M*. *tuberculosis* infection. Data from animal models have revealed conflicting evidence of a protective role of MR1T cells against *M*. *tuberculosis* disease in vivo. MR1^–/–^ mice were shown to have comparable survival to WT mice following infection with *M*. *tuberculosis* H37Rv. Further, 5-OP-RU vaccination failed to protect against TB disease despite robust expansion of MAIT cells in the lung (42–44). Intratracheal administration of 5-OP-RU in nonhuman primates led to functional MAIT cell exhaustion rather than expansion (45). However, 5-OP-RU treatment of mice during the chronic phase of infection was effective in reducing the bacterial burden in an IL-17A–dependent manner (43). Intravenous BCG vaccination of nonhuman primates was associated with sterilizing protection against *M*. *tuberculosis* challenge, and robust expansion of MAIT cells in the lungs was observed when compared with aerosol or intradermal BCG vaccination (46). These studies did not investigate the association between specific MR1T clonotypes and protection from *M*. *tuberculosis* disease. In our manuscript, we provide evidence that enriched MR1T clonotypes recognize mycobacteria-infected cells and bind to MR1-5-OP-RU tetramer. A limitation of these data is that they do not establish a link between recognition of infected cells in vitro and clearance of infection and host protection. Whether MR1T clonotypes enriched in RSTRs perform effector functions in vivo that are of importance in resistance is unclear. In addition, the breadth of MR1-presented antigens recognized by enriched clonotypes and the implications of this in protection require further elucidation (24).

In summary, our findings advance fundamental knowledge of MR1T cell clonotypic diversity in the context of a longitudinal human cohort. The specific associations we report with resistance to *M*. *tuberculosis* infection in humans are notable in light of several studies with negative findings of MAIT cells in *M*. *tuberculosis* disease animal models. Future work might focus on developing animal models of TB “resistance” in which the role of MR1T cells can be probed more specifically. At the same time, considerable effort will be required to discover new MR1 ligands and determine whether antigen-specific MR1T cell responses can mediate clearance of *M*. *tuberculosis* and underlie the RSTR phenotype.

## Methods

### Sex as a biological variable

Our cohort is balanced for sex to ensure an equal number of male and female individuals were included in both arms of the study.

### Study participants

The full details of the parent clinical study, including enrollment and sample collection, have been previously described (6). Briefly, African household contacts of sputum culture–positive cases of pulmonary TB were enrolled as part of the Kawempe Community Health Study conducted in Kampala, Uganda, between 2002 and 2012 (see Supplemental Table 1 for further details of participants). Contacts were sputum culture negative at enrollment and had no radiological evidence of active *M*. *tuberculosis* infection. Enrolled individuals were longitudinally profiled for signs of latent *M*. *tuberculosis* infection by TST (Mantoux method, 0.1 mL of 5 tuberculin units of purified protein derivative, Tubersol; Connaught Laboratories) over a 2-year observation period. TST positivity was defined as an induration > 10 mm for HIV^–^ individuals and > 5 mm for HIV^+^ individuals. Overall, 2,585 individuals were enrolled, and 10.7% of this group (*n* = 198) were persistently TST negative over a 2-year follow-up. Between 2014 and 2017, 691 individuals from this original household contact study were retraced, of which 441 (63.8%) were successfully reidentified and willing to participate in a subsequent longitudinal follow-up study. The median time between the initial study and retracing was 9.5 years. Retraced individuals completed 3 QuantiFERON-TB Gold (QFT) assays over 2 years. At their final visit, individuals were also tested by TST. A definite classification was assigned if TST assay results (5 from the original study and 1 at the end of the retracing study) and 3 QFT assays from the retracing study were concordantly negative or positive (RSTR or LTBI, respectively). PBMCs were isolated from whole blood by Ficoll-Hypaque density centrifugation and cryopreserved until use.

### Generation of tetramers

All tetramers were generated as published previously (25, 47). Biotinylated CD1b monomers were provided by the NIH Tetramer Core Facility (Emory University, Atlanta, Georgia, USA). GMM was a gift from D. Branch Moody (Harvard Medical School, Boston, Massachusetts, USA), and synthetic diacylated sulfoglycolipid (Ac_2_SGL) was provided by Adriaan Minnaard (University of Groningen, Groningen, the Netherlands). For CD1b monomer loading, GMM or Ac_2_SGL was dried down under a nitrogen stream and then sonicated into 50 mM sodium citrate buffer at pH 4, containing 0.25% 3-[(3-cholamidopropyl) dimethylammonio]-1-propanesulfonate (MilliporeSigma) for 2 minutes at 37°C. CD1b monomer was added with the resulting lipid suspensions at either 100-fold or 40-fold molar excess of CD1b monomer. The monomer and lipid suspensions were subsequently incubated at 37°C for 2 hours, with vortexing every 30 minutes. Following incubation, the solution was neutralized to pH 7.4 using 6 μL of 1 M Tris pH 9. Loaded lipid monomers were tetramerized by the addition of 1.25 molar equivalents of fluorophore-conjugated streptavidin (either BV421 conjugated for CD1b-GMM, BioLegend; or PE-conjugated for Ac_2_GSL, Life Technologies), assuming a 4:1 ratio of biotin to streptavidin was needed. Streptavidin was added over the course of 2 hours, 1/10th of the needed volume streptavidin was added, the vial was mixed, and the vial was incubated for 10 minutes before another volume was added. Tetramers were filtered for aggregates through a SpinX column (MilliporeSigma), then stored at 4°C until use. Mock-loaded CD1b tetramers were generated by an analogous process without the addition of exogenous lipids.

PBS-57–loaded (α-GalCer) and mock-loaded human CD1d monomers were provided by the NIH Tetramer Core Facility. Tetramers were prepared as previously described (48). Briefly, 10 μL of stock 2 mg/mL CD1d–α-GalCer was combined with 2.6 μL of streptavidin BV650 (BD Biosciences) every 10 minutes for 100 minutes until a final volume of 26 μL was reached. The tetramer was filtered through a SpinX column (MilliporeSigma) to remove aggregates and then stored at 4°C until use. MR1- 5-OP-RU tetramer was obtained from the NIH Tetramer Core Facility and used as provided.

### Flow cytometry

PBMCs were thawed and washed in warmed RPMI 1640 (Gibco) supplemented with 10% FBS (Hyclone) and 2 μL/mL Benzonase (MilliporeSigma). PBMCs were then resuspended at a density of 2 million cells/mL in RPMI/10% FBS and allowed to rest overnight at 37°C in humidified incubators supplemented with 5% CO_2_. The following day, the PBMCs were enumerated using the Guava easyCyte. One million cells/well were plated into a 96-well, U-bottom plate with up to 4 wells plated per sample. Cells were blocked with human serum (Valley Biomedical) prepared in FACS buffer 1× PBS (Gibco) supplemented with 0.2% BSA (MilliporeSigma) mixed 1:1 for 15 minutes at 4°C. A 13-color, multiparameter flow cytometry panel targeting DURT, lineage, and memory populations was used to characterize cells from each individual. Cells were centrifuged at 700*g* for 3 minutes; resuspended in 50 μL of FACS buffer containing CD1b-GMM, CD1b-AM Ac_2_SGL, CD1d–α-GalCer, MR1-5-OP-RU, and mock-loaded tetramers; and incubated at room temperature for 60 minutes. The cells were then washed twice with PBS and stained with Live/Dead Fixable Green Dead Cell Stain Kit (Life Technologies) per the manufacturer’s instructions. Following a 15-minute incubation at room temperature, the cells were washed twice in PBS and then labeled with anti-CD3 ECD (clone UCHT1; Beckman Coulter), anti-CD4 APC Alx750 (clone 13B8.2; Beckman Coulter), anti-CD8α PerCP Cy5.5 (clone SK1; BD Biosciences), anti-CD45RA BUV737 (clone HI100; BD Biosciences), anti-CCR7 BUV395 (clone 150503; BD Biosciences), anti-TRAV1-2 BV510 (clone 3C10; BioLegend), anti-Pan-γδ PE-Vio770 (clone 11f2; Miltenyi Biotec), and anti-Vδ2 BV711 (clone B6; BioLegend) for 30 minutes at room temperature. The optimal titers of all antibodies and tetramers were determined prior to use. After 2 final washes in FACS buffer, the cells were fixed in 1% paraformaldehyde (Electron Microscopy Sciences) and acquired on an LSRFortessa (BD Biosciences) equipped with blue (488 nm), green (532 nm), red (628 nm), violet (405 nm), and ultraviolet (355 nm) lasers using standardized good clinical laboratory practice procedures to minimize the variability of data generated.

### Cell sorting

PBMCs were thawed in warmed RPMI (10% FBS with 2 μL/mL benzonase) and washed twice in 5 mL at 300*g* for 10 minutes. After washing, cells were resuspended in 96-well, U-bottom plates and blocked prior to staining with 1 μg/mL anti-CD40 (clone HB14, Miltenyi Biotec) for 30 minutes at 37°C. Following blocking, cells were stained with MR1-5-OP-RU tetramer as described above for 60 minutes at room temperature. Samples were washed in FACS buffer to remove unbound tetramer prior to surface staining with anti-TCRαβ APC (clone IP26) and anti-CD7 FITC (clone CD7-6B7, BioLegend) for 30 minutes at 4°C. Samples were washed and resuspended in 500 μL of FACS buffer before sorting using the BD Biosciences FACSAria II Cell Sorter equipped with blue (488 nm), red (641 nm), and violet (407 nm) lasers. CD7^+^TCRβ^+^MR1-5-OP-RU^+^, live, intact singlets were sorted for downstream analysis (Supplemental Figure 1A).

### CITE-Seq and scTCR-Seq

For CITE-Seq experiments, cells were stained with oligo-tagged antibodies: anti-CD3 (clone UCHT1), anti-CD4 (clone RPA-T4), anti-CD8a (clone RPA-T8), anti-CD56 (clone QA17A16), anti-CD45RO (clone UCHL1), anti-CD69 (clone FN50), anti-CD27 (clone O323), anti-CD39 (clone A1), anti-CD244 (clone C1.7), anti-CD127 (IL-7Ra; clone A019D5), anti-CD38 (clone HIT2), anti-CD71 (clone CY1G4), anti-CXCR3 (clone G025H7), anti-CD196 (clone G034E3), anti-CD161 (clone HP-3G10), anti–HLA-DR (clone L243), anti-CX3CR1 (clone K0124E1), anti-CD81 (clone 5A6), anti-CD28 (clone CD28.2), anti-CD26 (clone BA5b), anti-TCR Va7.2 (clone C0581), anti-Human Hashtag 1 (CD45; clone LNH-94), anti-Human Hashtag 2 (CD45; clone LNH-94), anti-Hashtag 3 (CD45; clone LNH-94), and anti-Hashtag 4 (CD45; clone LNH-94). All antibodies were obtained from BioLegend. Cells were stained with the sorting antibody cocktail and MR1-5-OP-RU tetramer for 30 minutes at 4°C and sorted as described above.

Single-cell library preparation for surface protein, mRNA, and TCRs was performed using the Chromium Single Cell V(D)J Reagent Kit v1.1 (10x Genomics). The TCR V(D)J region was specifically enriched in a separate library preparation using the Chromium Single-Cell V(D)J Enrichment Kit (10x Genomics). Cell suspensions were combined with barcoded, single-cell 5′ gel beads and loaded onto Chromium Next GEM Chip G (10x Genomics) at a limiting dilution such that a single bead and a single cell are partitioned into a sphere. Libraries were sequenced by Illumina sequencing (NextSeq 500/550 platform). Sequence alignment was performed using Cell Ranger (10x Genomics). All mRNA and V(D)J reads were aligned to the GRCh38 human reference genome.

### TCRα/δ immunosequencing

For each sample (*n* = 40), DNA was extracted from PBMCs using the QIAGEN DNeasy Blood and Tissue kit. DNA was quantified using TapeStation (Agilent). TCRα/δ chains were sequenced using the immunoSEQ high-throughput sequencing platform (Adaptive Biotechnologies) (49). For each sample, multiplex PCR was used to amplify rearranged VDJ sequences followed by high-throughput sequencing using Illumina technologies. PCR amplification bias was minimized by internal controls in the immunoSEQ assay (50). Raw data were exported to R from the Adaptive Biotechnologies immunoSEQ Analyzer.

### Construction of MR1-restricted TCR cassette

MR1-restricted TCRs associated with RSTR status in TCRα/δ immunosequencing were selected for experimental validation. For each TCR, nucleotide sequences for the TRA and TRB V- and J-genes were obtained from IMGT (51). CDR3α and CDR3β nucleotide sequences obtained directly from sequencing data were added and confirmed to be in frame. TCR constant regions were substituted with modified murine homologs to facilitate assessment of TCR surface expression by flow cytometry. Complete TRA and TRB polypeptides were linked via a porcine teschovirus-1 P2A linker (52). Final constructs were codon optimized and subcloned into lentiviral vector pRRLSIN.cPPT.PGK-GFP.WPRE, resulting in 2 vectors named pRRL.KLSF and pRRL.QLIW.

### Generation of lentivirus

HEK293T cells (CRL-3216, ATCC) were seeded in a 100 mm tissue culture dish at a density of 2 × 10^6^ cells per dish. Cultures were propagated at 37°C until 80% confluence was reached. Cultures were maintained in DMEM (Gibco) supplemented with 10% FBS (Cytiva Life Sciences), 100 U/mL penicillin/100 mg/mL streptomycin (Thermo Fisher Scientific), 200 mM l-glutamine (Gibco), and 0.1 mM MEM Non-Essential Amino Acids (Gibco). Culture media were replaced 2 hours prior to transfection.

HEK293T cell cultures were transfected with either 10 μg pRRL-KLSF or pRRL.QLIW plasmid, 5 μg pCI-VSVG envelope plasmid, and 5 μg of a psPAX2 packaging vector (53). Plasmids were mixed with Fugene6 reagent at a ratio of 1:12 (Promega) to ensure efficient transfection. Transfected cells were cultured overnight, and media were completely replaced the following morning. Culture supernatants containing lentivirus were harvested every 12 hours from 36 hours posttransfection for a total of 3 collections per culture. Supernatants were incubated at 4°C with Lenti-X Concentrator (Clontech) overnight. Precipitated lentivirus was resuspended and stored at –80°C until use.

### Transduction of Jurkat cell lines

Each TCR was separately expressed in a Jurkat E6–1 cell line with Nur77-mNeonGreen reporter, which has been previously described (54). Briefly, mNeonGreen was integrated downstream of TCR signaling into the *NR4A1* locus using CRISPR/Cas9 technology. Endogenous TCR expression was knocked out, and Jurkat cells were clonally sorted based on TCRβ and CD3 surface expression. In addition, constitutive CD8αβ expression was added via integration into the actin locus using CRISPR/Cas9. Jurkat cell lines were maintained in RPMI 1640 medium (Gibco) supplemented with 10% FBS, 100 U/mL penicillin/100 mg/mL streptomycin, and 200 mM l-glutamine.

For each TCR, 1 million Jurkat-mNeonGreen cells were transduced in a 48-well plate with 200 μL of either pRRL.KLSF or pRRL.QLIW lentivirus at an estimated 5 infectious units per cell (MOI of 5). Polybrene was included in each transduction well at a final concentration of 4 μg/mL (MilliporeSigma). Cultures were incubated overnight at 37°C with 5% CO_2_ and washed in PBS the following morning to remove excess lentivirus. Cultures were maintained for 7 days posttransduction and assessed for TCR surface expression by staining for anti-mouse TCRβ-APC (clone H57-597, BD Biosciences) and anti-human CD3-BUV395 (clone UCHT1, BD Biosciences). An average transduction efficiency of 38% and 42% was observed for pRRL.KLSF and pRRL.QLIW, respectively. CD3^+^TCRβ^+^ double-positive events were sorted for purity and further propagated in culture as above for downstream experiments.

### Coculture of Jurkat and infected antigen-presenting cells

Jurkat transductants were cocultured with an A549 cell line (ATCC, CCL-185) infected with BCG Russia expressing mCherry (provided by David Sherman, University of Washington, Seattle, Washington). A549 cells were cultured in base medium F12/K (Gibco) supplemented with 10% FBS, 100 U/mL penicillin/100 mg/mL streptomycin, and 200 mM l-glutamine. One day before infection, A549 cells were seeded into a 96-well, U-bottom plate at a density of 2 × 10^4^ cells/well in antibiotic-free media. Cultures were incubated overnight to allow cell adherence to the plate. A549 cell lines were then infected with BCG grown to log-phase at an estimated 30 infectious units or 10 infectious units per cell for 12 hours. Culture media were changed 12 hours postinfection to remove remaining extracellular bacteria. TCR-expressing Jurkat cells were then cocultured with infected A549 cells at a target/effector ratio of 10:1 for 16 hours. TCR signaling was assessed via Nur77 expression using flow cytometry. Cells were stained for viability (Live Dead Zombie Yellow, Life Technologies).

### Statistics

#### Flow cytometry.

Spillover compensation and initial quality assessment were performed using FlowJo version 9.9.6 (TreeStar Inc.). Supplemental Figure 1B provides a representative gating strategy for identification of DURT cell subsets. T cell subset counts, as a proportion of either CD3^+^, live, intact singlets or CD3^+^MR1-5-OP-RU^+^, live, intact singlets, were exported and analyzed in the R programming environment. All samples had acceptable viability and frequencies of CD3^+^ events and were included in downstream analysis. Subset frequencies were compared between groups using a Wilcoxon rank sum test.

#### CITE-Seq.

All data analysis was performed in the R programming environment using Seurat workflows. Hashtag oligos (HTOs) were transformed using a centered log-ratio transformation (CLR) applied with the ScaleData function in Seurat 4.0 (30). Cells were demultiplexed into original donor samples based on enrichment of HTOs using the HTODemux function in Seurat. Next, the probability of a cell being degraded was estimated using a probabilistic mixture model framework implemented using the miQC package (55). Percentage of mitochondrial reads and total gene count per cell were used as input variables. A posterior probability threshold of 0.75 was used to remove compromised cells. Initial analyses of each sequencing batch identified batch effects in both gene and protein expression datasets. Batches were integrated using canonical correlation analysis in Seurat before further downstream analysis (56). Variability in gene expression sequencing depth between samples was corrected for by normalization using the function scTransform in Seurat (57). Antibody-derived tag counts for each marker were transformed using CLR before downstream analysis. Gene and surface protein expression were used to define clusters using the WNN workflow in Seurat (resolution = 0.5) (30). Differential expression of genes between clusters was performed in Seurat using Wilcoxon rank sum tests. These tests were performed on the RNA assay using FindConservedMarkers in Seurat using batch as a grouping variable. A gene was differentially expressed for a given cluster if it was significantly changed between that cluster and the remaining data (FDR = 0.05 with Bonferroni correction for multiple testing). Highly variable genes in the data set (default selection of top 3,000 genes was used) were used as input for principal component analysis (PCA). The default number of principal components was computed (*n* = 50). Other dimensionality reductions such as UMAP were performed using principal components as input. UMAP and PCA were both implemented in Seurat.

#### scTCR-Seq.

Reads were mapped to the TRA and TRB loci using Cell Ranger software. Contigs were filtered for productive rearrangements with in-frame CDR3 sequences. Additional filtering was applied to include only viable cells (as identified in scRNA-Seq pre-processing) and barcodes with a paired TCRα and TCRβ chain. Finally, contigs were collapsed to produce a data frame where each row represented a single barcode using the scRepertoire package (58). Global repertoire metrics (evenness, diversity, number of unique clonotypes) were computed in R using the Immunarch package in Zenodo. Identification of biochemically similar TCRs was performed using TCRdist in Python (29, 59). In TCRdist, CDR loops that contact the pMHC complex are concatenated into a single string. These are then used as input to compute a distance matrix between all unique TCR clonotypes in our data set. The distance measure is a similarity-weighted Hamming distance with a gap penalty applied in cases where CDR sequences are different lengths. The CDR3 sequence is given a higher weight given its importance in pMHC recognition. The TCRdist distance matrix was then used for clustering (hierarchical clustering using the hclust function in the stats package v3.6.1 in base R) and dimensionality reduction (multidimensional scaling using the cmdscale function in the stats package).

#### Graph-versus-graph analysis.

Relationships between single-cell gene expression profile and TCR clonotype were assessed using CoNGA (33). Briefly, similarity graphs were constructed in gene expression and TCR space, respectively. In the gene expression graph, nodes represented cells, and edges represented correlation in gene expression. In the TCR graph, nodes represented cells, and edges represented pairwise TCR similarity based on the TCRdist distance metric (described above). Only cells that passed pre-processing described above were included in the analysis. Neighborhood overlap between the 2 graphs was evaluated. The number of vertices connected to each clonotype in the gene expression and TCR graphs was individually counted, and a significance score was generated based on whether the number of overlapping connections was greater than expected by chance.

#### TCRα/δ immunosequencing.

Raw data were exported from Adaptive Biotechnologies’ immunoSEQ Analyzer and analyzed in R. Clonotypes that were in frame and had both a V-gene and a J-gene assignment with a CDR3α amino acid length of more than 5 were included in further analyses. Following pre-processing, contingency tables of TCRα or TCRδ clonotypes were statistically assessed for enrichment in either RSTR or LTBI donors using 2-sided Fisher’s exact tests and a nominal *P* value cutoff of 0.01. In addition, significantly enriched clonotypes identified in immunosequencing data were parsed for known MR1T clonotypes using our scTCR-Seq as a reference. Clonotype matching based on nucleotide sequence yielded no hits, so matches based on CDR3α amino acid sequence and TCRα V- and TCRα J-gene usage were used.

### Study approval

The retracing study protocol was approved by the National AIDS Research Committee, the Uganda National Council on Science and Technology (both in Kampala, Uganda), and the institutional review board at University Hospitals Cleveland Medical Center in Cleveland, Ohio, USA. All study participants gave written informed consent, as approved by the institutional review boards of the participating institutions.

### Data availability

Scripts for all analyses are available at https://github.com/seshadrilab/MR1_RSTRs.git (commit ID 1d51d75). Multimodal scRNA-Seq data and clonotype matrices can be downloaded from NCBI GEO (accession no. GSE264592). The flow cytometry data supporting this publication are available from ImmPort (https://www.immport.org) under study workspace ID 6982. TCRα/δ sequencing data are available from the zenodo website (https://zenodo.org/records/10999748). Supporting Data Values for all figures are provided.

## Author contributions

DLC performed the data integration, analysis, and visualization. DLC and CS wrote the paper with contributions from all authors. Flow cytometry data were acquired by EDL and KKQY. MSA performed MR1T cell sorting. KKQY, MSA, and SL performed multimodal scRNA-Seq. HMK, CMS, TRH, and WHB facilitated access to clinical specimens. EWN oversaw scRNA-Seq and advised on MR1T cell sorting strategy. PB performed CoNGA, including visualization. MTS performed the initial data cleaning. AGC and ECW provided Nur77-expressing Jurkat cell lines.

## Supplementary Material

Supplemental data

Supporting data values

## Figures and Tables

**Table 1:**
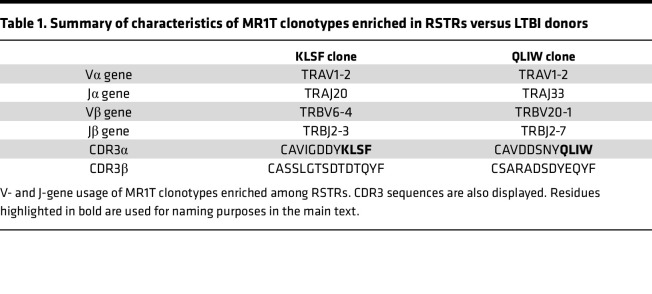
Summary of characteristics of MR1T clonotypes enriched in RSTRs versus LTBI donors

## Version 1 05/08/2024

Electronic publication
