# Supplementary material for: MR1-restricted T cell clonotypes are associated with “resistance” to Mycobacterium tuberculosis infection
Source: JCI Insight. 2024 May 8;9(9):e166505. doi: 10.1172/jci.insight.166505 (PMC11141901; doi:10.1172/jci.insight.166505)
Supplement: Supplemental data [file jciinsight-9-166505-s172.pdf]

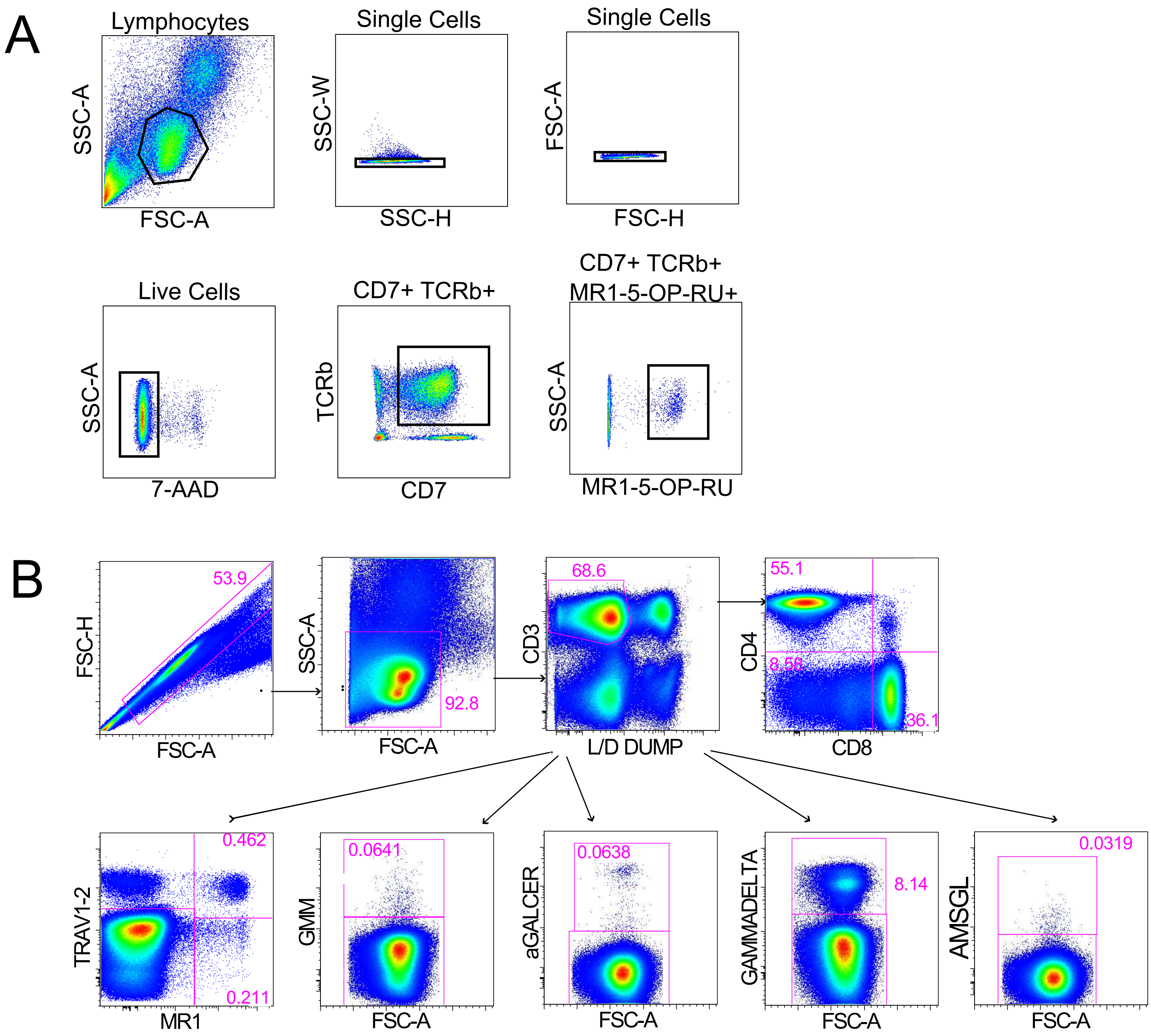

Supplementary Figure 1. (A) Gating scheme identifying donor-unrestricted T-cells in peripheral blood of RSTR and LTBI donors (B) Sorting scheme identifying CD7+ TCRab+ MR1-5-OP-RU+ cells in peripheral blood of RSTR and LTBI donors.

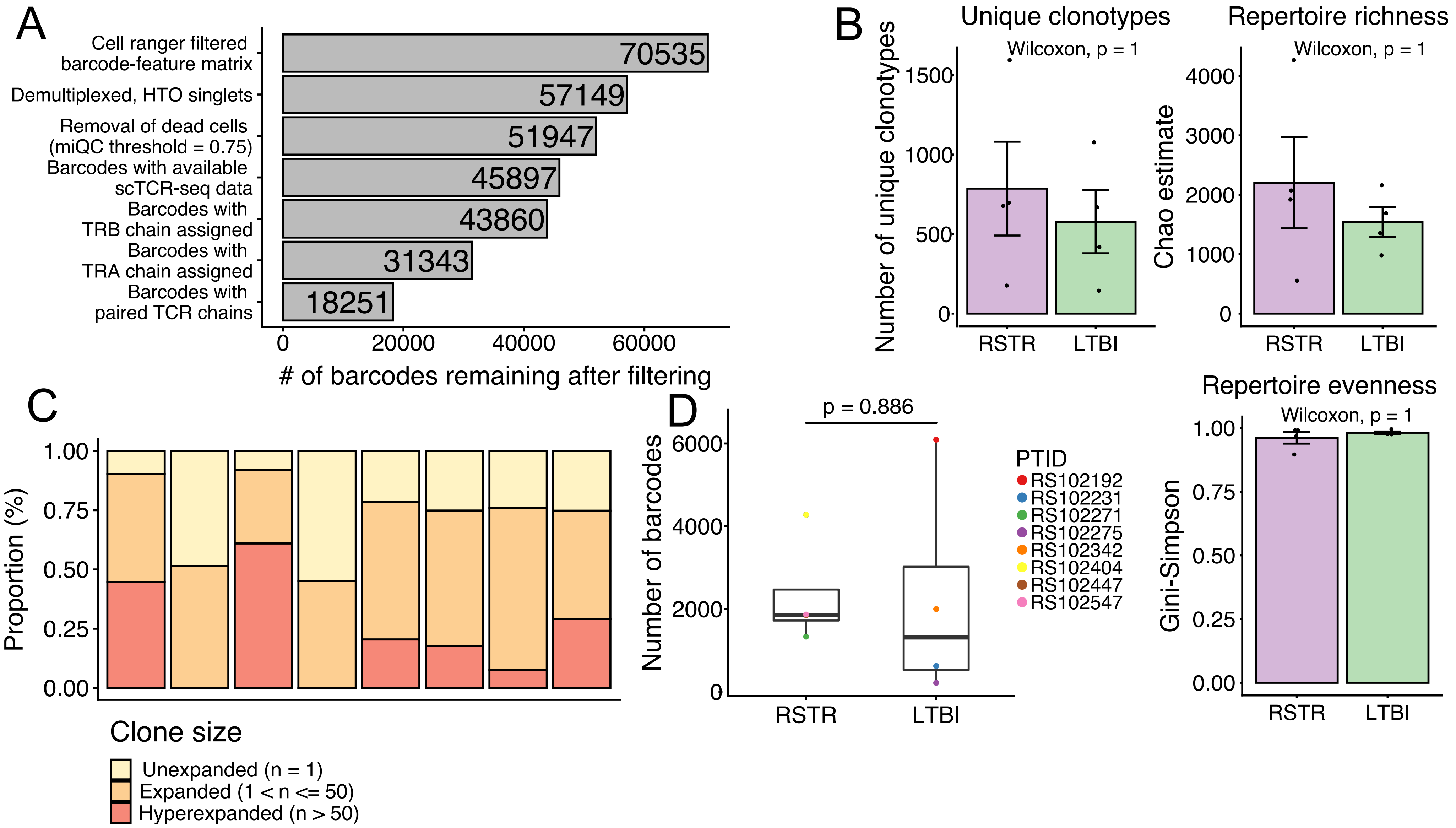

Supplementary Figure 2. (A) Summary of CITE-seq QC filtering (B) Global diversity metrics summarizing the MR1T repertoire. (C) The proportion of unexpanded (n = 1), expanded (1<n<=50) and hyperexpanded (n > 50) MR1T clonotypes within each donor. A clonotype is defined based on nucleotide sequence (D) The number of barcodes per sample, compared between TB groups. Statistical testing was performed using a Wilcoxon Rank Sum test.

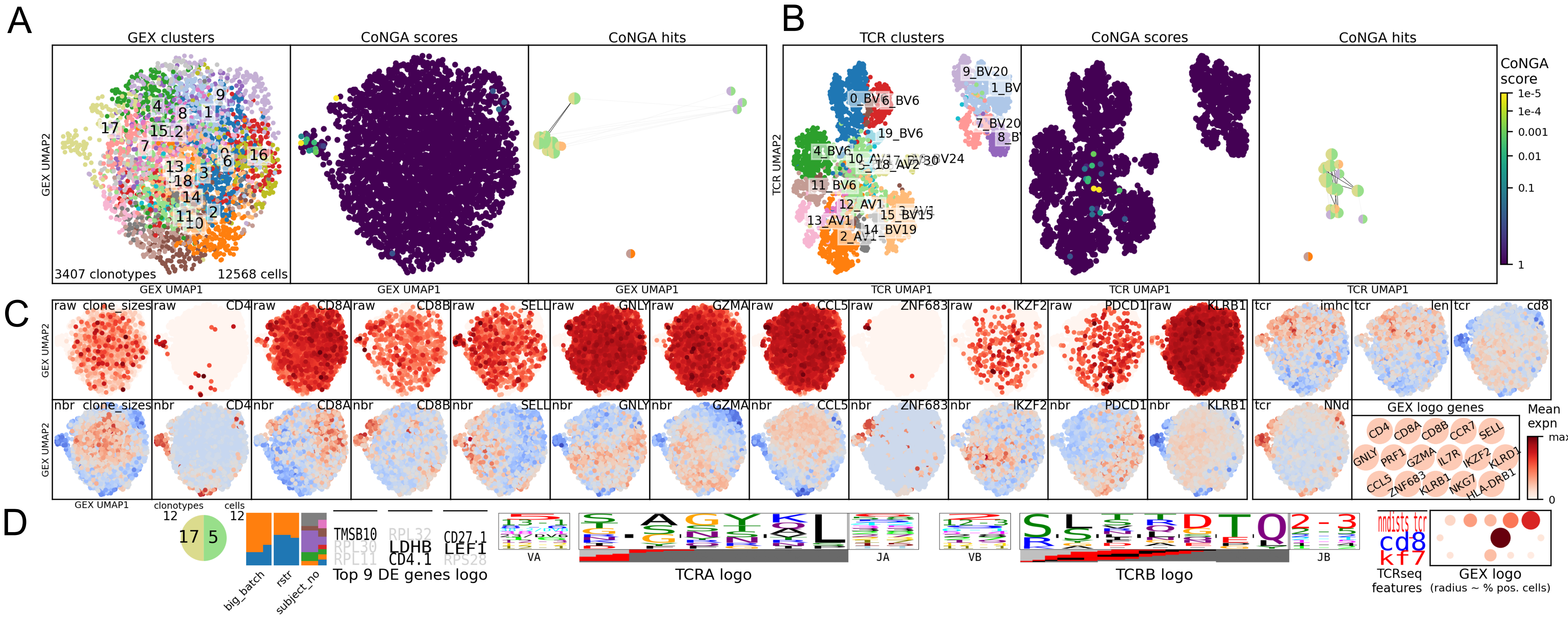

Supplementary Figure 3. Graph-vs-graph analysis of MR1T clonotypes (A) UMAP of MR1 T clonotypes based on similarity in gene expression. Panels are coloured left to right by gene expression cluster, CoNGA score – indicating the clonotypes with significant overlap between their gene expression and TCR similarity – and CoNGA hits (clonotypes with a significant CoNGA score). (B) UMAP of MR1 T clonotypes based on similarity in TCR CDR3 sequence. Panels are coloured left to right by TCR cluster, CoNGA score and significant CoNGA scores. (C) gene expression UMAP colored by gene expression (D) Consensus logos for CDR3[illegible] and CDR3[illegible] sequences, V and J gene usage for all clones with a significant CoNGA score.

Supplementary Table 1. Summary of study participant demographic characteristics

	Subjects not included in this study	Subjects included in this study	p-value	Significance
Number of donors (n)	277	50	NA	NS
Gender (% male)	49.20	56.80	0.23	NS
Current Age (years, median)	22.00	21.00	0.76	NS
Adult Epidemiological Risk Score[A] (mean)	6.45	6.49	0.72	NS
Duration of follow-up (years, median)	9.99	9.16	0.03	*

The included donors represent all individuals across flow cytometry, immunosequencing and CITE-seq experiments (n = 50). Values presented are either medians or means as indicated. Gender was compared using Chi-square test, all other variables were compared using Mann-Whitney test; p-values indicate the results of these tests. Significance thresholds are as follows: * <= 0.05, ** <= 0.01, *** <= 0.001. [A] Details of risk scoring criteria have been previously published (see Ref. 7 for information)

Supplementary Table 2. Summary of differentially expressed genes identified between subsets of MR1-restricted T-cells.

Cluster	Gene	Batch 1 p-value	Batch 1 Avg Log2FC	Batch 1 % of cells expressing marker in selected cluster	Batch 1 % of cells expressing marker in remaining data	Batch 1 adjusted p-value	Batch 2 p-value	Batch 2 Avg Log2FC	Batch 2 % of cells expressing marker in selected cluster	Batch 2 % of cells expressing marker in remaining data	Batch 2 adjusted p-value	Combined p-value
0	RPS28	0.00	1.48	1.00	1.00	0.00	0.39	8.96	1.00	1.00	1.00	0.00
0	RPL17	0.00	0.61	0.76	0.67	0.00	0.00	1.20	0.99	0.98	1.00	0.00
0	MALAT1	0.00	30.36	1.00	1.00	0.00	0.00	86.83	1.00	1.00	1.00	0.00
0	SLC38A1	0.00	0.49	0.60	0.52	1.00	0.02	0.39	0.45	0.45	1.00	0.00
0	NSA2	0.00	0.26	0.56	0.51	1.00	0.00	0.32	0.72	0.68	1.00	0.00
0	RPL29	0.00	1.48	1.00	1.00	1.00	0.29	0.61	1.00	1.00	1.00	0.00
0	STK17B	0.00	0.44	0.64	0.57	1.00	0.09	0.99	0.59	0.57	1.00	0.00
0	PARP8	0.00	0.39	0.57	0.51	1.00	0.20	0.78	0.52	0.50	1.00	0.00
0	TPM4	0.00	0.28	0.24	0.20	1.00	0.02	0.26	0.29	0.26	1.00	0.00
0	UBB	0.00	2.77	0.87	0.82	1.00	0.26	0.62	0.89	0.88	1.00	0.00
0	FUS	0.00	1.42	0.64	0.60	1.00	0.00	0.48	0.64	0.61	1.00	0.00
0	MYBL1	0.00	0.37	0.37	0.35	1.00	0.89	0.68	0.37	0.37	1.00	0.00
0	EIF3H	0.00	0.33	0.68	0.61	1.00	0.41	0.27	0.74	0.73	1.00	0.00
0	DDX24	0.00	0.52	0.67	0.62	1.00	0.60	0.57	0.64	0.63	1.00	0.01
0	RPL23	0.01	0.46	0.74	0.72	1.00	0.75	2.44	0.83	0.83	1.00	0.02
0	IRF1	0.08	4.63	0.55	0.52	1.00	0.39	0.45	0.65	0.64	1.00	0.15
0	GLTSCR2	0.13	0.94	0.81	0.79	1.00	0.47	0.83	0.85	0.84	1.00	0.25
0	RP11-796E2.4	0.96	0.55	0.15	0.15	1.00	0.25	0.31	0.17	0.15	1.00	0.44
1	AHNAK	0.00	1.16	0.42	0.52	0.00	0.07	3.12	0.54	0.56	1.00	0.00
2	PRF1	0.00	1.66	0.71	0.61	0.00	0.00	3.27	0.80	0.74	0.00	0.00
2	AC092580.4	0.00	1.67	0.40	0.29	0.00	0.00	0.80	0.78	0.72	0.00	0.00
2	IL2RG	0.00	4.71	0.77	0.72	0.00	0.00	1.69	0.87	0.83	0.00	0.00

2	G3BP2	0.00	0.57	0.58	0.45	0.00	0.00	0.43	0.57	0.52	0.09	0.00
2	GUK1	0.00	0.52	0.80	0.71	0.00	0.00	0.46	0.84	0.81	0.52	0.00
2	HCST	0.00	1.95	0.90	0.85	0.00	0.05	2.98	0.96	0.95	1.00	0.00
2	RPL36A	0.00	1.32	0.77	0.69	0.00	0.88	0.50	0.80	0.80	1.00	0.00
2	CTSA	0.00	0.53	0.31	0.24	0.12	0.00	0.32	0.39	0.33	0.00	0.00
2	ADGRE5	0.01	0.25	0.39	0.35	1.00	0.00	1.63	0.42	0.37	0.00	0.00
2	HLA-F	0.00	0.71	0.66	0.59	0.00	0.88	0.39	0.63	0.63	1.00	0.00
2	SPOCK2	0.00	3.34	0.83	0.77	0.00	0.00	7.04	0.85	0.83	0.48	0.00
2	DNAJB6	0.00	1.22	0.53	0.48	0.03	0.00	1.02	0.61	0.57	0.00	0.00
2	TRBV6-4	0.00	0.63	0.31	0.24	0.01	0.00	1.11	0.20	0.16	0.46	0.00
2	PRDM1	0.00	0.32	0.27	0.21	0.01	0.00	0.54	0.31	0.26	0.34	0.00
2	BCL3	0.00	0.35	0.38	0.32	1.00	0.00	0.40	0.52	0.47	0.04	0.00
2	TRBV6-1	0.00	0.54	0.07	0.11	0.05	0.16	1.34	0.12	0.14	1.00	0.00
2	ITM2B	0.00	0.98	0.90	0.87	0.08	0.00	1.26	0.95	0.94	1.00	0.00
2	SURF4	0.00	0.31	0.40	0.33	0.08	0.00	0.29	0.39	0.36	1.00	0.00
2	CANX	0.00	0.31	0.45	0.36	0.08	0.39	0.35	0.42	0.42	1.00	0.00
2	CSRNP1	0.00	0.54	0.60	0.55	0.30	0.00	0.33	0.44	0.39	1.00	0.00
2	DDX21	0.00	0.44	0.41	0.36	0.39	0.00	0.29	0.48	0.44	1.00	0.00
2	NR4A2	0.00	1.07	0.70	0.65	1.00	0.00	4.97	0.62	0.60	0.68	0.00
2	UQCRB	0.00	4.47	0.82	0.77	1.00	0.76	0.43	0.90	0.90	1.00	0.00
2	SRSF3	0.00	1.01	0.70	0.66	1.00	0.18	1.03	0.66	0.65	1.00	0.00
2	MYH9	0.00	2.44	0.58	0.51	1.00	0.74	2.27	0.55	0.54	1.00	0.00
2	TRBC2	0.00	0.70	0.53	0.50	1.00	0.04	0.65	0.58	0.54	1.00	0.00
2	LITAF	0.00	0.63	0.94	0.92	1.00	0.22	1.16	0.89	0.90	1.00	0.00
2	BCLAF1	0.00	0.28	0.44	0.40	1.00	0.54	0.29	0.41	0.41	1.00	0.00
2	SNHG8	0.00	0.63	0.58	0.52	1.00	0.62	0.47	0.73	0.74	1.00	0.00
2	TRBC1	0.00	2.36	0.29	0.24	1.00	0.00	0.90	0.37	0.33	1.00	0.00

2	CDC37	0.00	0.33	0.49	0.43	1.00	0.09	0.51	0.58	0.58	1.00	0.00
2	ODF2L	0.01	0.28	0.34	0.31	1.00	0.00	0.27	0.43	0.40	1.00	0.00
2	FURIN	0.00	0.28	0.17	0.12	1.00	0.04	0.54	0.18	0.16	1.00	0.00
2	HNRNPDL	0.00	0.52	0.84	0.80	1.00	0.00	0.70	0.89	0.87	1.00	0.00
2	ERN1	0.02	0.28	0.40	0.36	1.00	0.00	0.33	0.43	0.41	1.00	0.01
2	SEC61B	0.05	0.86	0.61	0.57	1.00	0.00	0.36	0.70	0.67	1.00	0.01
2	FOSL2	0.01	0.63	0.37	0.33	1.00	0.33	1.31	0.36	0.35	1.00	0.01
2	EIF3K	0.03	0.38	0.80	0.77	1.00	0.78	0.78	0.89	0.88	1.00	0.05
2	HSP90B1	0.04	0.49	0.62	0.60	1.00	0.57	0.53	0.70	0.70	1.00	0.09
2	S100A11	0.65	0.27	0.22	0.22	1.00	0.16	0.45	0.31	0.29	1.00	0.29
2	TTC39C	0.33	0.27	0.31	0.29	1.00	0.20	0.30	0.40	0.39	1.00	0.35
3	NOSIP	0.00	1.20	0.69	0.45	0.00	0.00	2.22	0.79	0.52	0.00	0.00
3	LGALS3	0.00	1.78	0.41	0.16	0.00	0.00	1.65	0.35	0.18	0.00	0.00
3	CCR6	0.00	0.44	0.40	0.31	1.00	0.00	1.02	0.43	0.24	0.00	0.00
3	TOB1	0.00	3.17	0.53	0.35	0.00	0.00	0.93	0.44	0.28	0.00	0.00
3	TIMP1	0.00	1.07	0.26	0.14	0.00	0.00	1.16	0.38	0.22	0.00	0.00
3	IFITM2	0.00	0.91	0.71	0.63	0.00	0.00	0.40	0.89	0.83	0.00	0.00
3	ZFP36L2	0.00	7.51	1.00	1.00	0.00	0.00	13.82	1.00	0.99	0.00	0.00
3	ARHGAP15	0.00	0.63	0.50	0.34	0.00	0.00	1.05	0.53	0.41	0.00	0.00
3	S100A11	0.04	0.36	0.23	0.22	1.00	0.00	0.74	0.41	0.27	0.00	0.00
3	DDX3Y	0.00	0.39	0.21	0.10	0.00	0.00	0.39	0.18	0.08	0.00	0.00
3	BLK	0.00	1.39	0.29	0.12	0.00	0.00	0.57	0.21	0.10	0.00	0.00
3	CRIP1	0.01	0.31	0.33	0.31	1.00	0.00	4.20	0.66	0.53	0.00	0.00
3	KLRC1	0.00	0.35	0.11	0.05	0.00	0.00	0.41	0.14	0.06	0.00	0.00
3	MYL12A	0.00	1.05	0.91	0.84	0.00	0.00	1.61	0.97	0.94	0.00	0.00
3	GPR183	0.00	0.74	0.27	0.16	0.00	0.00	0.87	0.26	0.15	0.00	0.00
3	ALOX5AP	0.01	0.55	0.59	0.56	1.00	0.00	0.40	0.74	0.66	0.00	0.00

3	DAD1	0.00	0.70	0.42	0.29	0.00	0.00	0.37	0.52	0.45	0.01	0.00
3	KDSR	0.00	0.34	0.20	0.11	0.00	0.00	0.39	0.27	0.18	0.00	0.00
3	SOCS3	0.00	0.72	0.28	0.20	0.00	0.00	0.34	0.39	0.29	0.00	0.00
3	RBMS1	0.00	0.59	0.44	0.31	0.00	0.00	0.36	0.47	0.38	0.00	0.00
3	RCAN3	0.00	0.47	0.23	0.16	0.44	0.00	0.26	0.28	0.19	0.00	0.00
3	SELL	0.10	0.65	0.19	0.17	1.00	0.00	1.34	0.30	0.21	0.00	0.00
3	CD53	0.00	0.40	0.51	0.45	1.00	0.00	0.48	0.56	0.48	0.00	0.00
3	MFSD10	0.00	0.33	0.28	0.18	0.00	0.00	0.47	0.34	0.27	0.00	0.00
3	CSTB	0.00	0.41	0.28	0.20	0.77	0.00	0.32	0.44	0.35	0.00	0.00
3	GPSM3	0.00	0.62	0.62	0.55	0.48	0.00	0.47	0.75	0.70	0.00	0.00
3	KRTCAP2	0.00	0.63	0.52	0.43	0.00	0.00	0.31	0.67	0.62	0.01	0.00
3	TMEM123	0.00	0.50	0.50	0.40	0.00	0.00	0.27	0.49	0.42	0.00	0.00
3	CCDC85B	0.00	0.31	0.48	0.40	1.00	0.00	0.61	0.56	0.50	0.00	0.00
3	GDI2	0.00	0.27	0.31	0.25	1.00	0.00	0.34	0.37	0.32	0.01	0.00
3	C10orf54	0.00	0.35	0.45	0.36	0.01	0.00	0.35	0.48	0.44	1.00	0.00
3	FKBP11	0.00	0.27	0.50	0.41	1.00	0.00	0.59	0.55	0.48	0.01	0.00
3	RP11-290D2.6	0.00	4.65	0.37	0.28	0.10	0.02	1.17	0.15	0.13	1.00	0.00
3	SERP1	0.00	0.64	0.74	0.69	0.12	0.01	5.61	0.74	0.71	1.00	0.00
3	PRNP	0.00	0.53	0.45	0.36	0.17	0.00	0.29	0.46	0.43	1.00	0.00
3	RPL36A	0.00	1.33	0.78	0.70	0.33	0.35	0.31	0.80	0.80	1.00	0.00
3	PAK2	0.00	0.29	0.42	0.38	1.00	0.00	0.36	0.47	0.43	0.68	0.00
3	CDKN1B	0.00	0.53	0.48	0.41	1.00	0.18	0.69	0.49	0.48	1.00	0.00
3	HNRNPU	0.00	0.31	0.52	0.47	1.00	0.30	1.76	0.52	0.50	1.00	0.00
3	MTDH	0.22	0.33	0.47	0.43	1.00	0.00	0.47	0.55	0.50	1.00	0.00
3	NFKBIZ	0.00	2.20	0.47	0.39	1.00	0.00	0.50	0.30	0.26	1.00	0.00
3	BAX	0.01	0.51	0.36	0.30	1.00	0.00	1.74	0.46	0.42	1.00	0.00

3	FAM107B	0.00	0.36	0.35	0.29	1.00	0.02	1.90	0.53	0.53	1.00	0.00
3	RPS27L	0.01	0.35	0.28	0.24	1.00	0.00	1.15	0.39	0.36	1.00	0.01
3	EDF1	0.01	0.38	0.67	0.62	1.00	0.32	0.41	0.76	0.74	1.00	0.02
3	EIF4A2	0.07	0.30	0.58	0.54	1.00	0.56	0.46	0.58	0.57	1.00	0.13
3	SUN2	0.07	0.25	0.52	0.48	1.00	0.09	0.57	0.58	0.56	1.00	0.14
3	BTG1	0.18	18.88	0.99	1.00	1.00	0.42	1.27	1.00	0.99	1.00	0.33
4	RPS4X	0.41	17.65	1.00	1.00	1.00	0.00	12.89	1.00	1.00	0.00	0.00
4	RPS25	0.00	2.08	1.00	0.99	1.00	0.00	15.97	1.00	1.00	0.00	0.00
4	RPL10	0.47	19.49	1.00	1.00	1.00	0.00	44.07	1.00	1.00	0.00	0.00
4	RPL30	0.48	3.69	1.00	1.00	1.00	0.00	13.70	1.00	1.00	0.00	0.00
4	RPS15A	0.46	0.61	1.00	1.00	1.00	0.00	12.45	1.00	1.00	0.00	0.00
4	RPL11	0.64	13.64	1.00	1.00	1.00	0.00	12.91	1.00	1.00	0.00	0.00
4	RPS13	0.45	0.40	1.00	1.00	1.00	0.00	13.67	1.00	1.00	0.00	0.00
4	HLA-E	0.31	6.00	1.00	0.99	1.00	0.00	17.44	1.00	1.00	0.00	0.00
4	RPS29	0.00	0.91	0.97	0.95	0.41	0.00	20.92	1.00	1.00	0.00	0.00
4	RPL29	0.78	3.56	1.00	1.00	1.00	0.00	4.64	1.00	1.00	0.00	0.00
4	RPLP0	0.04	1.44	1.00	1.00	1.00	0.00	3.50	1.00	1.00	0.00	0.00
4	COX4I1	0.33	1.03	0.93	0.92	1.00	0.00	8.88	0.99	0.97	0.00	0.00
4	RPL12	0.26	9.09	1.00	1.00	1.00	0.00	10.56	1.00	1.00	0.00	0.00
4	UBA52	0.15	4.89	0.98	0.98	1.00	0.00	1.58	1.00	0.99	0.00	0.00
4	MT-ND3	0.00	8.62	0.98	0.94	0.00	0.00	0.96	1.00	1.00	0.00	0.00
4	PNRC1	0.83	2.16	0.92	0.92	1.00	0.00	3.77	0.97	0.92	0.00	0.00
4	RPS20	0.21	1.17	0.90	0.88	1.00	0.00	6.91	0.95	0.93	0.00	0.00
4	KMT2E	0.21	0.48	0.68	0.68	1.00	0.00	1.29	0.78	0.69	0.00	0.00
4	PIK3R1	0.87	0.65	0.75	0.75	1.00	0.00	1.53	0.82	0.74	0.00	0.00
4	RPL7	0.84	1.15	0.96	0.95	1.00	0.00	0.55	0.99	0.98	0.00	0.00
4	LAPTM5	0.15	0.50	0.90	0.89	1.00	0.00	2.44	0.96	0.93	0.00	0.00

4	HNRNPDL	0.88	0.67	0.81	0.81	1.00	0.00	1.86	0.91	0.87	0.00	0.00
4	SARAF	0.29	2.50	0.95	0.94	1.00	0.00	0.73	0.98	0.97	0.00	0.00
4	PIK3IP1	0.79	0.31	0.54	0.53	1.00	0.00	0.89	0.62	0.52	0.00	0.00
4	TAPBP	0.99	0.72	0.52	0.52	1.00	0.00	0.90	0.64	0.56	0.00	0.00
4	REL	0.02	0.69	0.48	0.46	1.00	0.00	0.43	0.60	0.50	0.00	0.00
4	COMMD6	0.10	0.66	0.69	0.72	1.00	0.00	1.25	0.93	0.88	0.00	0.00
4	ARL4C	0.12	0.42	0.77	0.75	1.00	0.00	6.51	0.85	0.81	0.00	0.00
4	DDX24	0.23	0.71	0.63	0.62	1.00	0.00	0.66	0.70	0.63	0.00	0.00
4	CCNI	0.49	0.62	0.87	0.87	1.00	0.00	3.93	0.93	0.92	0.00	0.00
4	EIF5	0.02	0.83	0.54	0.50	1.00	0.00	0.44	0.63	0.53	0.00	0.00
4	RELB	0.00	0.26	0.38	0.33	1.00	0.00	0.35	0.48	0.36	0.00	0.00
4	COX7C	0.54	0.97	0.84	0.83	1.00	0.00	3.30	0.96	0.95	0.00	0.00
4	FYN	0.14	0.50	0.58	0.54	1.00	0.00	1.60	0.65	0.58	0.00	0.00
4	RORA	0.06	2.30	0.67	0.63	1.00	0.00	0.49	0.72	0.64	0.00	0.00
4	ARF1	0.42	0.70	0.71	0.71	1.00	0.00	0.71	0.85	0.79	0.00	0.00
4	RPL34	0.51	2.59	1.00	1.00	1.00	0.00	9.38	1.00	1.00	0.00	0.00
4	CDC42	0.77	0.43	0.60	0.60	1.00	0.00	0.33	0.72	0.67	0.00	0.00
4	G3BP2	0.05	0.43	0.45	0.47	1.00	0.00	0.66	0.61	0.52	0.00	0.00
4	EML4	0.00	2.31	0.75	0.69	0.03	0.00	0.46	0.71	0.64	0.00	0.00
4	C12orf57	0.04	15.83	0.70	0.66	1.00	0.00	3.19	0.83	0.78	0.00	0.00
4	TGFB1	0.00	1.29	0.63	0.56	0.06	0.00	4.50	0.68	0.60	0.00	0.00
4	PNISR	0.66	0.35	0.43	0.43	1.00	0.00	0.28	0.65	0.57	0.00	0.00
4	EMD	0.28	0.32	0.52	0.51	1.00	0.00	0.42	0.67	0.60	0.00	0.00
4	MT-ND2	0.00	25.53	0.96	0.93	0.00	0.00	8.29	0.99	0.98	0.00	0.00
4	SRSF3	0.02	0.82	0.69	0.66	1.00	0.00	0.35	0.72	0.65	0.00	0.00
4	GZMM	0.12	0.60	0.67	0.64	1.00	0.00	2.71	0.81	0.78	0.00	0.00
4	SLC38A1	0.32	0.35	0.52	0.53	1.00	0.00	0.32	0.54	0.44	0.00	0.00

4	ELF1	0.01	0.65	0.55	0.50	1.00	0.00	0.35	0.56	0.47	0.00	0.00
4	HNRNPUL1	0.24	3.65	0.53	0.52	1.00	0.00	0.43	0.58	0.55	0.00	0.00
4	NOP58	0.00	0.59	0.45	0.39	1.00	0.00	0.30	0.50	0.43	0.00	0.00
4	SERINC1	0.49	0.28	0.32	0.33	1.00	0.00	0.51	0.40	0.34	0.00	0.00
4	TAF1D	0.23	0.26	0.42	0.39	1.00	0.00	0.66	0.52	0.44	0.00	0.00
4	CST7	0.00	0.32	0.95	0.93	1.00	0.00	3.63	0.97	0.95	0.00	0.00
4	SYNE2	0.94	11.68	0.52	0.52	1.00	0.00	3.18	0.61	0.53	0.00	0.00
4	MAPK1IP1L	0.45	0.25	0.46	0.46	1.00	0.00	0.43	0.57	0.51	0.00	0.00
4	ARGLU1	0.07	0.43	0.46	0.47	1.00	0.00	0.46	0.58	0.50	0.02	0.00
4	PDIA3	0.85	2.44	0.64	0.65	1.00	0.00	0.75	0.74	0.70	0.02	0.00
4	CEBPD	0.00	3.37	0.65	0.58	0.02	0.00	3.20	0.55	0.51	1.00	0.00
4	ATF7IP2	0.80	0.46	0.38	0.37	1.00	0.00	0.66	0.50	0.44	0.03	0.00
4	IL7R	0.11	4.73	0.99	0.98	1.00	0.00	13.94	0.99	0.98	0.04	0.00
4	RP11-160E2.6	0.05	1.24	0.34	0.32	1.00	0.00	0.34	0.35	0.28	0.05	0.00
4	ETS1	0.60	0.71	0.56	0.55	1.00	0.00	0.78	0.63	0.57	0.06	0.00
4	SYAP1	0.01	0.30	0.42	0.37	1.00	0.00	0.35	0.39	0.32	0.08	0.00
4	RHOG	0.31	0.33	0.40	0.38	1.00	0.00	0.78	0.55	0.48	0.11	0.00
4	SNU13	0.00	0.25	0.50	0.46	1.00	0.00	0.38	0.61	0.56	0.12	0.00
4	SF1	0.03	0.93	0.61	0.61	1.00	0.00	0.72	0.70	0.66	0.13	0.00
4	RNF125	0.78	0.29	0.45	0.45	1.00	0.00	0.42	0.48	0.42	0.15	0.00
4	SEC61G	0.74	5.00	0.54	0.53	1.00	0.00	0.33	0.75	0.69	0.30	0.00
4	WIPF1	0.02	0.43	0.62	0.58	1.00	0.00	0.46	0.72	0.67	0.59	0.00
4	UFC1	0.87	0.39	0.39	0.40	1.00	0.00	1.03	0.59	0.53	0.63	0.00
4	IDS	0.00	0.68	0.52	0.48	1.00	0.00	0.64	0.54	0.48	0.82	0.00
4	CSNK1D	0.02	1.36	0.30	0.26	1.00	0.00	0.27	0.36	0.29	1.00	0.00
4	ATP6V0C	0.35	0.72	0.51	0.51	1.00	0.00	0.26	0.53	0.47	1.00	0.00

4	NR1H2	0.00	0.28	0.29	0.25	1.00	0.00	0.29	0.34	0.29	1.00	0.00
4	MX1	0.02	1.25	0.17	0.14	1.00	0.00	0.27	0.17	0.12	1.00	0.00
4	MACF1	0.14	0.31	0.20	0.21	1.00	0.00	0.27	0.29	0.24	1.00	0.00
4	BHLHE40	0.80	0.38	0.56	0.55	1.00	0.00	0.98	0.54	0.47	1.00	0.00
4	TRBC1	0.14	1.87	0.27	0.24	1.00	0.00	0.53	0.36	0.34	1.00	0.00
4	EIF4G2	0.31	1.60	0.49	0.51	1.00	0.00	0.34	0.55	0.49	1.00	0.00
4	CD83	0.62	0.40	0.12	0.13	1.00	0.00	0.30	0.18	0.14	1.00	0.00
4	BRD2	0.96	0.31	0.49	0.48	1.00	0.00	0.31	0.46	0.40	1.00	0.00
4	STAT1	0.25	1.50	0.14	0.15	1.00	0.00	0.92	0.19	0.15	1.00	0.00
4	ROMO1	0.11	0.26	0.27	0.24	1.00	0.00	0.28	0.49	0.43	1.00	0.00
4	IFITM1	0.33	13.47	0.92	0.91	1.00	0.00	0.47	0.98	0.97	1.00	0.01
4	FLT3LG	0.67	0.27	0.36	0.36	1.00	0.00	0.57	0.53	0.48	1.00	0.01
4	USP36	0.43	0.39	0.37	0.36	1.00	0.01	0.33	0.34	0.30	1.00	0.01
4	IFITM2	0.24	3.30	0.63	0.64	1.00	0.01	1.00	0.85	0.83	1.00	0.02
4	DDX18	0.69	0.35	0.22	0.22	1.00	0.02	0.40	0.40	0.36	1.00	0.05
4	TRBV6-2	0.14	1.58	0.12	0.10	1.00	0.03	2.54	0.19	0.22	1.00	0.06
4	PRF1	0.42	3.07	0.63	0.62	1.00	0.11	1.53	0.78	0.75	1.00	0.20
4	TRBV6-4	0.13	1.92	0.23	0.26	1.00	0.83	0.67	0.17	0.17	1.00	0.24
5	CD8B	0.00	0.77	0.35	0.19	0.00	0.00	0.62	0.51	0.20	0.00	0.00
5	CD6	0.03	1.63	0.47	0.43	1.00	0.00	0.40	0.58	0.53	0.14	0.00
5	ZFP36	0.00	29.71	0.98	0.97	1.00	0.00	14.55	0.93	0.93	0.88	0.00
5	DNAJA1	0.01	0.40	0.48	0.40	1.00	0.04	0.48	0.42	0.39	1.00	0.01
5	RP11-51J9.5	0.01	0.33	0.24	0.18	1.00	0.92	0.78	0.33	0.33	1.00	0.02
5	PRRC2C	0.08	0.27	0.62	0.57	1.00	0.92	0.27	0.61	0.61	1.00	0.15
5	TNF	0.26	8.22	0.20	0.17	1.00	0.42	8.07	0.12	0.12	1.00	0.45
6	FOS	0.00	5.74	0.99	0.98	1.00	0.00	58.58	0.96	0.94	0.00	0.00
6	MYLIP	0.00	0.39	0.51	0.38	0.62	0.09	0.55	0.36	0.34	1.00	0.00

6	RPS10	0.00	1.07	0.61	0.50	1.00	0.58	3.26	1.00	1.00	1.00	0.00
6	FTL	0.00	2.69	0.98	0.98	1.00	0.83	80.29	0.99	0.99	1.00	0.00
6	DNAJB1	0.00	0.29	0.76	0.67	1.00	0.14	12.68	0.60	0.58	1.00	0.00
6	OSTF1	0.00	0.32	0.44	0.33	1.00	0.28	0.58	0.45	0.43	1.00	0.00
6	CD44	0.00	0.54	0.76	0.68	1.00	0.62	0.76	0.73	0.72	1.00	0.00
6	GABARAP	0.00	0.29	0.55	0.46	1.00	0.36	3.70	0.68	0.65	1.00	0.00
6	TOMM7	0.00	1.03	0.87	0.84	1.00	0.91	3.06	0.96	0.96	1.00	0.00
6	GPX4	0.00	0.31	0.46	0.42	1.00	0.94	0.43	0.54	0.53	1.00	0.00
6	GATA3	0.00	1.48	0.33	0.27	1.00	0.46	3.22	0.34	0.36	1.00	0.01
6	ZYX	0.01	0.30	0.35	0.29	1.00	0.18	0.63	0.41	0.38	1.00	0.03
6	RELB	0.02	0.59	0.41	0.33	1.00	0.86	0.86	0.37	0.37	1.00	0.03
6	GZMM	0.03	0.26	0.72	0.65	1.00	0.22	0.36	0.79	0.78	1.00	0.05
6	BTG2	0.03	0.28	0.65	0.58	1.00	0.09	3.88	0.58	0.54	1.00	0.05
6	SNHG8	0.03	0.53	0.57	0.53	1.00	0.37	1.19	0.75	0.73	1.00	0.06
6	CSDE1	0.05	0.32	0.59	0.52	1.00	0.06	0.55	0.59	0.55	1.00	0.11
6	EIF2S2	0.06	0.26	0.24	0.19	1.00	0.14	0.34	0.35	0.33	1.00	0.12
6	LINC00936	0.14	0.33	0.18	0.18	1.00	0.06	0.80	0.25	0.22	1.00	0.12
6	ACAP1	0.07	0.38	0.41	0.38	1.00	0.18	0.77	0.51	0.54	1.00	0.14
6	TSPO	0.08	0.26	0.32	0.29	1.00	0.82	6.08	0.47	0.47	1.00	0.15
6	ST13	0.08	0.26	0.45	0.40	1.00	0.22	0.54	0.57	0.56	1.00	0.15
6	ZFAS1	0.09	0.34	0.63	0.57	1.00	0.55	0.64	0.69	0.69	1.00	0.18
6	UXT	0.10	0.59	0.55	0.57	1.00	0.19	0.26	0.66	0.68	1.00	0.18
6	MCL1	0.25	6.01	0.57	0.54	1.00	0.10	1.53	0.47	0.44	1.00	0.20
6	TYROBP	0.52	3.74	0.18	0.16	1.00	0.10	15.60	0.15	0.18	1.00	0.20
6	ABLIM1	0.24	1.64	0.26	0.24	1.00	0.11	0.36	0.34	0.31	1.00	0.21
6	TAF1D	0.32	0.25	0.37	0.40	1.00	0.11	0.60	0.47	0.44	1.00	0.22
6	BHLHE40	0.13	2.03	0.61	0.55	1.00	0.51	1.02	0.49	0.48	1.00	0.25

6	SSR4	0.72	1.29	0.76	0.75	1.00	0.17	0.99	0.85	0.84	1.00	0.31
6	H3F3B	0.21	3.56	1.00	1.00	1.00	0.27	38.44	1.00	1.00	1.00	0.38
6	SF3B2	0.98	0.28	0.51	0.50	1.00	0.28	0.26	0.57	0.57	1.00	0.48
7	CCL5	0.00	24.34	0.99	0.97	0.00	0.00	37.33	0.99	0.98	0.00	0.00
7	CD52	0.00	49.99	0.89	0.89	0.00	0.00	25.59	0.98	0.96	0.00	0.00
7	PFN1	0.00	126.18	0.97	0.97	0.00	0.00	203.22	0.99	0.99	0.00	0.00
7	MT-CO1	0.00	38.69	1.00	1.00	0.00	0.50	28.16	1.00	1.00	1.00	0.00
7	GZMH	0.00	0.46	0.19	0.08	0.00	0.00	35.27	0.19	0.07	0.00	0.00
7	NKG7	0.00	2.23	0.99	0.97	0.00	0.00	27.22	0.99	0.99	0.00	0.00
7	GAPDH	0.00	38.20	0.96	0.95	0.00	0.00	167.17	0.98	0.97	0.00	0.00
7	C12orf75	0.00	0.42	0.43	0.33	0.70	0.00	10.57	0.56	0.42	0.00	0.00
7	HSP90AA1	0.00	6.51	0.87	0.82	0.00	0.00	38.46	0.92	0.87	0.00	0.00
7	ACTB	0.00	286.33	1.00	1.00	0.03	0.00	581.23	1.00	1.00	0.00	0.00
7	GZMA	0.08	10.66	0.77	0.75	1.00	0.00	49.04	0.90	0.88	0.00	0.00
7	TMSB4X	0.00	151.66	0.99	0.99	0.00	0.00	192.96	1.00	1.00	0.00	0.00
7	COTL1	0.00	25.79	0.13	0.09	0.07	0.00	48.86	0.31	0.19	0.00	0.00
7	B2M	0.00	41.55	1.00	1.00	0.00	0.00	69.07	1.00	1.00	0.00	0.00
7	TMSB10	0.00	77.46	0.99	0.98	0.00	0.00	118.56	0.99	0.99	1.00	0.00
7	HLA-DPB1	0.00	0.28	0.27	0.19	1.00	0.00	0.83	0.34	0.23	0.00	0.00
7	GBP5	0.09	5.66	0.42	0.39	1.00	0.00	2.12	0.57	0.49	0.00	0.00
7	PSME2	0.09	8.64	0.53	0.51	1.00	0.00	20.02	0.70	0.65	0.00	0.00
7	SH3BGRL3	0.00	41.54	0.99	0.96	0.00	0.00	76.77	0.99	0.99	0.00	0.00
7	CFL1	0.00	28.73	0.96	0.91	1.00	0.00	86.46	0.97	0.96	0.00	0.00
7	SUB1	0.00	1.05	0.69	0.61	0.00	0.00	12.17	0.73	0.67	0.14	0.00
7	CORO1A	0.00	40.81	0.79	0.78	0.08	0.00	40.49	0.92	0.89	0.00	0.00
7	CD2	0.00	0.49	0.38	0.31	1.00	0.00	0.94	0.57	0.49	0.00	0.00
7	PSMC1	0.00	0.31	0.31	0.17	0.00	0.00	0.26	0.27	0.21	1.00	0.00

7	FLNA	0.00	20.67	0.60	0.47	0.00	0.00	1.33	0.59	0.54	1.00	0.00
7	H3F3A	0.00	13.08	0.84	0.82	1.00	0.00	13.91	0.93	0.91	0.00	0.00
7	LSP1	0.00	9.15	0.77	0.73	1.00	0.00	0.59	0.89	0.85	0.00	0.00
7	UQCR10	0.00	1.06	0.48	0.34	0.00	0.06	7.53	0.55	0.51	1.00	0.00
7	ITM2A	0.00	0.71	0.34	0.32	1.00	0.00	1.19	0.55	0.47	0.00	0.00
7	LITAF	0.00	0.29	0.94	0.92	0.00	0.96	4.47	0.90	0.90	1.00	0.00
7	IL32	0.00	58.82	0.99	0.98	1.00	0.00	73.38	1.00	1.00	0.00	0.00
7	PCBP1	0.00	2.80	0.76	0.68	0.00	0.22	0.56	0.85	0.86	1.00	0.00
7	MYL6	0.00	10.20	0.88	0.84	1.00	0.00	36.38	0.94	0.93	0.00	0.00
7	CALM2	0.00	0.81	0.64	0.51	0.00	0.00	2.74	0.66	0.61	1.00	0.00
7	CD69	0.00	4.35	0.88	0.83	0.49	0.00	4.59	0.88	0.84	0.00	0.00
7	YWHAB	0.00	4.43	0.74	0.67	0.00	0.00	6.14	0.82	0.75	0.52	0.00
7	STK17A	0.00	1.46	0.65	0.56	0.00	0.05	5.24	0.60	0.57	1.00	0.00
7	EVL	0.00	3.77	0.60	0.56	1.00	0.00	0.84	0.78	0.72	0.00	0.00
7	CCNH	0.00	0.41	0.51	0.39	0.00	0.00	0.34	0.41	0.34	1.00	0.00
7	PRKCH	0.00	0.52	0.53	0.42	0.00	0.00	0.34	0.51	0.42	1.00	0.00
7	HLA-B	0.00	51.48	1.00	1.00	0.00	0.00	17.13	1.00	1.00	0.11	0.00
7	RPS3A	0.00	2.14	1.00	1.00	0.01	0.00	2.89	1.00	1.00	1.00	0.00
7	ACTG1	0.00	88.97	0.91	0.93	0.02	0.00	172.93	0.97	0.96	0.01	0.00
7	MDH2	0.03	0.75	0.37	0.34	1.00	0.00	0.56	0.55	0.45	0.01	0.00
7	MSN	0.00	1.97	0.55	0.46	0.80	0.00	2.05	0.48	0.45	0.01	0.00
7	CALM1	0.00	11.28	0.98	0.96	0.01	0.00	23.54	0.98	0.99	1.00	0.00
7	PSME1	0.00	6.86	0.82	0.78	1.00	0.00	22.22	0.92	0.91	0.01	0.00
7	PTP4A2	0.00	0.56	0.45	0.38	0.01	0.00	0.33	0.46	0.39	0.36	0.00
7	ALDOA	0.00	0.50	0.82	0.71	0.01	0.04	11.27	0.87	0.83	1.00	0.00
7	GNB2	0.01	0.58	0.46	0.40	1.00	0.00	0.65	0.53	0.46	0.02	0.00
7	ARHGDIB	0.00	9.74	0.96	0.94	0.15	0.00	45.54	0.99	0.98	0.02	0.00

7	RHOA	0.00	5.28	0.83	0.76	0.02	0.00	7.36	0.88	0.84	1.00	0.00
7	ATP5E	0.00	1.41	0.86	0.81	0.12	0.00	19.23	0.98	0.97	0.03	0.00
7	MT-ND1	0.28	2.14	0.99	0.98	1.00	0.00	0.68	0.98	0.99	0.04	0.00
7	PSMB10	0.00	2.43	0.36	0.28	1.00	0.00	7.96	0.45	0.39	0.04	0.00
7	ARPC2	0.00	23.92	0.75	0.71	0.05	0.00	18.19	0.87	0.83	0.74	0.00
7	YWHAZ	0.00	6.79	0.93	0.87	0.05	0.04	3.72	0.91	0.88	1.00	0.00
7	KLRG1	0.00	1.76	0.56	0.51	0.06	0.00	0.93	0.73	0.66	0.07	0.00
7	ATP5B	0.00	1.82	0.62	0.54	0.17	0.00	3.98	0.68	0.63	0.06	0.00
7	GIMAP7	0.00	0.82	0.26	0.19	1.00	0.00	0.48	0.37	0.31	0.08	0.00
7	RPLP2	0.33	7.89	1.00	1.00	1.00	0.00	9.50	1.00	1.00	0.11	0.00
7	CAPZA1	0.00	0.29	0.38	0.26	0.13	0.35	0.90	0.35	0.33	1.00	0.00
7	CALR	0.04	2.39	0.61	0.57	1.00	0.00	15.71	0.68	0.64	0.13	0.00
7	HMGB1	0.00	15.15	0.86	0.81	0.14	0.00	20.05	0.90	0.87	0.24	0.00
7	GYPC	0.00	2.50	0.64	0.59	1.00	0.00	1.46	0.70	0.66	0.17	0.00
7	TMA7	0.00	7.12	0.83	0.81	0.19	0.03	18.89	0.97	0.96	1.00	0.00
7	LCP1	0.00	12.56	0.81	0.74	1.00	0.00	12.84	0.80	0.75	0.19	0.00
7	R3HDM4	0.01	0.31	0.34	0.27	1.00	0.00	0.38	0.40	0.34	0.19	0.00
7	PSMA4	0.00	2.73	0.23	0.20	1.00	0.00	1.93	0.34	0.25	0.25	0.00
7	ARPC1B	0.00	1.35	0.47	0.39	1.00	0.00	7.34	0.77	0.73	0.27	0.00
7	AATF	0.00	0.27	0.23	0.17	1.00	0.00	0.32	0.25	0.20	0.28	0.00
7	HNRNPA2B1	0.79	20.36	0.72	0.72	1.00	0.00	34.29	0.88	0.84	0.33	0.00
7	ARPC3	0.00	1.76	0.70	0.69	0.88	0.00	11.18	0.88	0.85	0.34	0.00
7	CD8A	0.00	0.85	0.81	0.72	0.35	0.12	0.99	0.71	0.70	1.00	0.00
7	DBI	0.00	0.67	0.34	0.35	1.00	0.00	6.20	0.58	0.50	0.49	0.00
7	CNN2	0.03	9.56	0.71	0.67	1.00	0.00	16.29	0.77	0.75	0.49	0.00
7	CAPZB	0.00	4.22	0.61	0.53	0.56	0.00	3.82	0.68	0.64	0.55	0.00
7	CIB1	0.00	0.41	0.52	0.41	0.55	0.13	0.41	0.50	0.46	1.00	0.00

7	ATP5A1	0.04	0.27	0.41	0.41	1.00	0.00	2.06	0.56	0.50	0.58	0.00
7	CD7	0.00	0.81	0.79	0.75	0.58	0.29	1.44	0.82	0.85	1.00	0.00
7	GNG2	0.00	0.40	0.49	0.41	1.00	0.00	0.63	0.52	0.43	0.65	0.00
7	PRR13	0.00	0.25	0.67	0.58	1.00	0.00	0.51	0.70	0.66	0.66	0.00
7	ITGB2	0.00	2.10	0.68	0.60	1.00	0.00	8.78	0.76	0.72	0.66	0.00
7	POMP	0.00	0.51	0.52	0.41	0.77	0.00	2.48	0.52	0.47	1.00	0.00
7	RAP1B	0.00	0.39	0.55	0.52	1.00	0.00	0.75	0.62	0.60	0.96	0.00
7	HNRNPC	0.00	0.75	0.60	0.52	1.00	0.06	1.35	0.66	0.65	1.00	0.00
7	CAP1	0.00	2.35	0.37	0.38	1.00	0.00	2.08	0.57	0.53	1.00	0.00
7	RBM3	0.00	1.92	0.85	0.82	1.00	0.01	6.38	0.87	0.86	1.00	0.00
7	ARHGDIA	0.00	0.53	0.64	0.55	1.00	0.43	0.63	0.65	0.63	1.00	0.00
7	GUK1	0.00	1.26	0.80	0.72	1.00	0.48	0.54	0.82	0.81	1.00	0.00
7	UQCRH	0.00	0.41	0.62	0.50	1.00	0.01	0.90	0.65	0.63	1.00	0.00
7	COX5A	0.00	0.37	0.40	0.32	1.00	0.00	9.37	0.53	0.45	1.00	0.00
7	LDHA	0.00	9.60	0.78	0.73	1.00	0.00	1.70	0.73	0.70	1.00	0.00
7	BSG	0.02	0.33	0.45	0.40	1.00	0.00	0.40	0.60	0.52	1.00	0.00
7	NDUFB11	0.00	0.67	0.55	0.48	1.00	0.74	7.03	0.61	0.59	1.00	0.00
7	MYH9	0.00	3.81	0.57	0.52	1.00	0.01	3.93	0.54	0.54	1.00	0.00
7	KDM5A	0.00	0.26	0.24	0.15	1.00	0.02	0.26	0.16	0.17	1.00	0.00
7	CD3G	0.00	1.29	0.56	0.50	1.00	0.00	1.48	0.68	0.64	1.00	0.00
7	GHITM	0.00	0.48	0.44	0.34	1.00	0.17	0.75	0.47	0.45	1.00	0.00
7	GMFG	0.00	3.70	0.69	0.61	1.00	0.00	0.83	0.82	0.77	1.00	0.00
7	PPP1CA	0.01	4.76	0.41	0.37	1.00	0.00	3.15	0.59	0.54	1.00	0.00
7	B3GNT2	0.07	0.26	0.33	0.28	1.00	0.00	0.30	0.33	0.27	1.00	0.00
7	SLC25A5	0.01	0.88	0.60	0.57	1.00	0.00	26.61	0.66	0.62	1.00	0.00
7	KLRD1	0.00	0.44	0.36	0.27	1.00	0.80	0.41	0.19	0.19	1.00	0.00
7	COX6C	0.00	1.04	0.67	0.61	1.00	0.00	4.27	0.81	0.76	1.00	0.00

7	TPM3	0.00	9.28	0.78	0.73	1.00	0.01	10.06	0.84	0.81	1.00	0.00
7	TAGAP	0.00	0.55	0.38	0.29	1.00	0.00	3.88	0.51	0.44	1.00	0.00
7	RAC2	0.21	10.32	0.69	0.69	1.00	0.00	2.54	0.85	0.81	1.00	0.00
7	SRP14	0.00	1.52	0.82	0.76	1.00	0.03	4.03	0.89	0.89	1.00	0.00
7	EPC1	0.11	0.26	0.59	0.54	1.00	0.00	0.73	0.63	0.58	1.00	0.00
7	CIRBP	0.00	3.41	0.92	0.91	1.00	0.03	0.47	0.96	0.96	1.00	0.00
7	MRPL54	0.05	0.26	0.36	0.31	1.00	0.00	0.26	0.45	0.38	1.00	0.00
7	PSMA7	0.00	1.41	0.50	0.45	1.00	0.00	2.30	0.53	0.51	1.00	0.00
7	ABRACL	0.14	2.53	0.31	0.26	1.00	0.00	0.52	0.43	0.35	1.00	0.00
7	DCTN3	0.00	0.33	0.35	0.26	1.00	0.01	0.99	0.37	0.33	1.00	0.00
7	ATP5L	0.00	5.61	0.84	0.81	1.00	0.02	14.37	0.93	0.93	1.00	0.00
7	TCEA1	0.00	0.26	0.42	0.32	1.00	0.01	0.42	0.45	0.40	1.00	0.00
7	C14orf166	0.09	0.25	0.41	0.35	1.00	0.00	2.57	0.49	0.44	1.00	0.00
7	GNG5	0.00	0.34	0.32	0.25	1.00	0.01	0.78	0.42	0.39	1.00	0.00
7	PPP1R18	0.01	0.32	0.23	0.18	1.00	0.00	0.37	0.36	0.28	1.00	0.00
7	WIPF1	0.00	0.64	0.67	0.58	1.00	0.00	1.30	0.73	0.67	1.00	0.00
7	NDUFB9	0.00	0.60	0.52	0.44	1.00	0.00	7.41	0.61	0.56	1.00	0.00
7	LDHB	0.09	2.05	0.62	0.59	1.00	0.00	22.55	0.70	0.67	1.00	0.00
7	PCBP2	0.00	2.91	0.95	0.94	1.00	0.12	16.57	0.97	0.97	1.00	0.00
7	RGS10	0.00	0.30	0.31	0.22	1.00	0.00	0.61	0.38	0.32	1.00	0.00
7	PSMB3	0.00	0.46	0.40	0.33	1.00	0.00	0.50	0.48	0.43	1.00	0.00
7	SLC25A3	0.00	0.55	0.64	0.57	1.00	0.08	10.55	0.67	0.64	1.00	0.00
7	CLDND1	0.00	0.26	0.18	0.13	1.00	0.07	0.47	0.22	0.23	1.00	0.00
7	PSMD8	0.00	0.33	0.38	0.32	1.00	0.00	2.87	0.41	0.36	1.00	0.00
7	CD55	0.00	0.33	0.45	0.36	1.00	0.80	0.41	0.34	0.33	1.00	0.00
7	C14orf2	0.00	0.50	0.42	0.32	1.00	0.05	1.27	0.57	0.52	1.00	0.00
7	GSTP1	0.00	0.63	0.39	0.33	1.00	0.00	13.57	0.44	0.40	1.00	0.00

7	ANXA5	0.00	0.33	0.33	0.27	1.00	0.00	4.99	0.43	0.38	1.00	0.00
7	ERH	0.00	0.44	0.42	0.33	1.00	0.10	3.85	0.48	0.44	1.00	0.00
7	EIF3D	0.01	0.33	0.43	0.41	1.00	0.00	0.30	0.60	0.54	1.00	0.00
7	PYHIN1	0.00	0.34	0.19	0.13	1.00	0.00	0.25	0.22	0.17	1.00	0.00
7	RSRC2	0.00	0.50	0.40	0.32	1.00	0.06	0.30	0.40	0.36	1.00	0.00
7	ETS1	0.00	2.06	0.62	0.55	1.00	0.98	0.50	0.58	0.58	1.00	0.00
7	HNRNPU	0.03	0.33	0.54	0.47	1.00	0.00	1.18	0.52	0.50	1.00	0.00
7	TRMT112	0.00	0.55	0.49	0.43	1.00	0.94	0.63	0.48	0.48	1.00	0.00
7	COX7B	0.00	0.78	0.47	0.38	1.00	0.05	3.44	0.56	0.51	1.00	0.00
7	HNRNPA3	0.00	1.47	0.64	0.57	1.00	0.04	2.24	0.67	0.63	1.00	0.00
7	CALM3	0.00	1.48	0.38	0.33	1.00	0.02	2.53	0.46	0.40	1.00	0.00
7	UQCRFS1	0.41	0.32	0.36	0.36	1.00	0.00	2.55	0.51	0.45	1.00	0.00
7	HMGN1	0.01	5.38	0.57	0.52	1.00	0.00	2.03	0.70	0.66	1.00	0.00
7	PTPN22	0.00	0.33	0.30	0.22	1.00	0.00	0.26	0.29	0.24	1.00	0.00
7	TUBA1B	0.00	0.35	0.54	0.45	1.00	0.00	71.99	0.48	0.45	1.00	0.00
7	WHSC1L1	0.01	0.50	0.38	0.32	1.00	0.00	0.37	0.41	0.36	1.00	0.00
7	ATP5G2	0.00	2.30	0.86	0.81	1.00	0.01	16.41	0.92	0.90	1.00	0.00
7	MYO1F	0.13	0.26	0.26	0.23	1.00	0.00	0.36	0.38	0.32	1.00	0.00
7	CTSD	0.02	0.37	0.32	0.30	1.00	0.00	0.33	0.42	0.37	1.00	0.00
7	COX6A1	0.00	2.52	0.63	0.54	1.00	0.00	3.42	0.73	0.70	1.00	0.00
7	ITGB7	0.17	2.76	0.23	0.20	1.00	0.00	0.38	0.33	0.29	1.00	0.00
7	NDUFAB1	0.00	0.25	0.31	0.23	1.00	0.00	0.31	0.36	0.33	1.00	0.00
7	PARK7	0.01	0.44	0.44	0.37	1.00	0.00	0.88	0.59	0.53	1.00	0.00
7	RPL22L1	0.00	0.30	0.53	0.49	1.00	0.03	1.56	0.58	0.56	1.00	0.00
7	SNRPB	0.00	0.32	0.50	0.42	1.00	0.23	3.41	0.55	0.52	1.00	0.00
7	EIF4H	0.00	0.32	0.44	0.35	1.00	0.19	0.86	0.40	0.40	1.00	0.00
7	HNRNPK	0.00	0.63	0.70	0.61	1.00	0.00	3.96	0.73	0.70	1.00	0.00

7	TOMM22	0.00	0.36	0.36	0.34	1.00	0.07	0.75	0.48	0.43	1.00	0.00
7	HNRNPF	0.00	1.40	0.41	0.35	1.00	0.02	1.47	0.42	0.40	1.00	0.00
7	COX8A	0.00	0.57	0.52	0.46	1.00	0.18	3.37	0.55	0.51	1.00	0.00
7	ATP5G3	0.05	0.45	0.53	0.47	1.00	0.00	3.17	0.64	0.57	1.00	0.00
7	PTMS	0.00	0.92	0.20	0.14	1.00	0.00	0.30	0.26	0.22	1.00	0.00
7	PDIA6	0.00	0.38	0.35	0.31	1.00	0.17	1.50	0.43	0.41	1.00	0.00
7	APMAP	0.13	0.70	0.34	0.31	1.00	0.00	0.51	0.47	0.43	1.00	0.00
7	SLC9A3R1	0.00	0.36	0.27	0.24	1.00	0.00	0.59	0.37	0.33	1.00	0.00
7	CXCR6	0.00	1.80	0.16	0.11	1.00	0.00	0.48	0.22	0.20	1.00	0.00
7	MAPK1IP1L	0.01	0.30	0.51	0.46	1.00	0.00	0.32	0.57	0.51	1.00	0.01
7	S100A4	0.04	41.54	0.99	0.98	1.00	0.00	11.27	1.00	1.00	1.00	0.01
7	ALOX5AP	0.00	0.51	0.64	0.56	1.00	0.10	0.95	0.65	0.67	1.00	0.01
7	SNRNP70	0.04	0.26	0.30	0.25	1.00	0.00	0.26	0.37	0.34	1.00	0.01
7	NDUFS5	0.00	0.50	0.54	0.48	1.00	0.04	0.36	0.59	0.55	1.00	0.01
7	FNBP4	0.00	0.26	0.43	0.34	1.00	0.27	0.30	0.38	0.36	1.00	0.01
7	H2AFV	0.00	8.79	0.48	0.41	1.00	0.81	1.23	0.47	0.48	1.00	0.01
7	RPL23A	0.00	4.46	0.99	0.99	1.00	0.07	5.61	0.99	1.00	1.00	0.01
7	SNRPD2	0.00	0.69	0.83	0.76	1.00	0.36	5.66	0.90	0.88	1.00	0.01
7	CYCS	0.00	0.39	0.53	0.48	1.00	0.01	0.40	0.56	0.50	1.00	0.01
7	HMGN2	0.00	27.60	0.65	0.61	1.00	0.08	69.13	0.73	0.70	1.00	0.01
7	BTF3	0.00	5.24	0.96	0.95	1.00	0.30	15.14	0.98	0.99	1.00	0.01
7	PRELID1	0.00	0.35	0.29	0.29	1.00	0.00	2.64	0.39	0.36	1.00	0.01
7	CASP4	0.00	0.57	0.32	0.27	1.00	0.01	1.52	0.41	0.37	1.00	0.01
7	SLC7A5	0.00	0.45	0.40	0.34	1.00	0.79	0.50	0.21	0.22	1.00	0.01
7	BIN1	0.00	0.29	0.36	0.28	1.00	0.07	0.41	0.36	0.37	1.00	0.01
7	VCP	0.00	1.38	0.45	0.39	1.00	0.01	0.46	0.46	0.42	1.00	0.01
7	HNRNPL	0.00	0.30	0.52	0.44	1.00	0.46	1.46	0.44	0.42	1.00	0.01

7	RPL7A	0.73	1.19	1.00	1.00	1.00	0.01	6.38	1.00	1.00	1.00	0.01
7	ATP5C1	0.01	0.45	0.36	0.31	1.00	0.16	0.84	0.39	0.38	1.00	0.01
7	PSMB9	0.01	1.23	0.50	0.46	1.00	0.03	0.89	0.68	0.64	1.00	0.01
7	CDK11A	0.01	0.45	0.24	0.18	1.00	0.05	0.28	0.24	0.20	1.00	0.01
7	OAZ1	0.01	7.25	0.79	0.73	1.00	0.40	3.42	0.72	0.73	1.00	0.01
7	PGAM1	0.01	0.28	0.40	0.34	1.00	0.25	7.94	0.48	0.45	1.00	0.01
7	TNFAIP8	0.11	0.27	0.28	0.26	1.00	0.01	0.29	0.40	0.37	1.00	0.01
7	LYAR	0.01	0.52	0.63	0.59	1.00	0.03	0.50	0.58	0.55	1.00	0.01
7	AP2S1	0.07	0.45	0.20	0.16	1.00	0.01	0.58	0.27	0.23	1.00	0.01
7	CD3D	0.06	1.94	0.64	0.67	1.00	0.01	3.09	0.84	0.81	1.00	0.01
7	CLIC1	0.06	2.15	0.60	0.59	1.00	0.01	1.69	0.73	0.72	1.00	0.02
7	CDC25B	0.01	0.50	0.20	0.17	1.00	0.03	1.93	0.29	0.25	1.00	0.02
7	AKNA	0.01	0.28	0.38	0.31	1.00	0.04	0.25	0.33	0.31	1.00	0.02
7	PTPRCAP	0.01	0.84	0.29	0.23	1.00	0.02	1.07	0.87	0.85	1.00	0.02
7	CELF2	0.01	0.69	0.45	0.38	1.00	0.53	0.63	0.48	0.46	1.00	0.02
7	TPR	0.01	0.90	0.30	0.24	1.00	0.09	0.34	0.30	0.27	1.00	0.02
7	SET	0.53	0.50	0.45	0.42	1.00	0.01	9.06	0.52	0.47	1.00	0.02
7	NDUFA4	0.01	0.31	0.67	0.60	1.00	0.24	1.12	0.78	0.75	1.00	0.03
7	CD37	0.01	10.55	0.79	0.73	1.00	0.90	0.28	0.83	0.83	1.00	0.03
7	COX7A2	0.02	2.16	0.67	0.60	1.00	0.02	0.38	0.77	0.75	1.00	0.03
7	DEK	0.02	0.52	0.37	0.35	1.00	0.98	10.45	0.46	0.46	1.00	0.03
7	PSMB1	0.04	0.28	0.52	0.49	1.00	0.02	3.27	0.64	0.59	1.00	0.04
7	YWHAQ	0.02	0.72	0.52	0.49	1.00	0.03	4.17	0.59	0.57	1.00	0.04
7	SERF2	0.10	17.35	0.91	0.89	1.00	0.02	29.22	0.96	0.96	1.00	0.04
7	TRBC2	0.02	2.04	0.54	0.50	1.00	0.10	1.77	0.59	0.55	1.00	0.04
7	CCDC167	0.02	0.29	0.17	0.13	1.00	0.06	0.35	0.29	0.26	1.00	0.04
7	HIGD2A	0.02	0.31	0.51	0.48	1.00	0.89	1.25	0.59	0.59	1.00	0.05

7	FOXP1	0.02	0.40	0.34	0.28	1.00	0.49	0.64	0.34	0.36	1.00	0.05
7	COX6B1	0.03	0.64	0.59	0.53	1.00	0.05	2.24	0.70	0.69	1.00	0.06
7	TRAV1-2	0.03	2.59	0.86	0.82	1.00	0.05	2.61	0.84	0.82	1.00	0.06
7	PPA1	0.03	0.35	0.30	0.24	1.00	0.57	0.84	0.38	0.38	1.00	0.06
7	ATPIF1	0.10	0.45	0.56	0.52	1.00	0.03	3.75	0.66	0.64	1.00	0.06
7	TRAC	0.03	0.54	0.15	0.12	1.00	0.24	0.53	0.16	0.15	1.00	0.06
7	PA2G4	0.03	0.34	0.34	0.30	1.00	0.20	0.81	0.45	0.42	1.00	0.06
7	RSRP1	0.03	2.14	0.51	0.46	1.00	0.14	0.40	0.49	0.50	1.00	0.06
7	HCLS1	0.04	0.40	0.56	0.51	1.00	0.03	1.46	0.68	0.67	1.00	0.06
7	HINT1	0.03	0.83	0.86	0.81	1.00	0.12	27.26	0.93	0.92	1.00	0.07
7	ANXA2	0.03	11.05	0.19	0.19	1.00	0.21	6.40	0.27	0.29	1.00	0.07
7	PSMA6	0.06	0.48	0.25	0.22	1.00	0.41	0.87	0.38	0.36	1.00	0.12
7	SNU13	0.06	0.44	0.53	0.46	1.00	0.20	0.38	0.60	0.56	1.00	0.12
7	HNRNPDL	0.07	0.61	0.82	0.81	1.00	0.56	1.66	0.89	0.87	1.00	0.13
7	ATP5F1	0.07	0.41	0.37	0.35	1.00	0.08	2.35	0.46	0.46	1.00	0.13
7	HSPD1	0.07	0.26	0.23	0.23	1.00	1.00	1.88	0.24	0.24	1.00	0.14
7	NDUFV2	0.08	0.83	0.32	0.28	1.00	0.81	0.35	0.37	0.38	1.00	0.16
7	OTUB1	0.14	0.38	0.30	0.26	1.00	0.08	0.87	0.35	0.35	1.00	0.16
7	PPIA	0.09	25.62	0.92	0.91	1.00	0.10	88.53	0.96	0.95	1.00	0.17
7	DRAP1	0.25	0.30	0.43	0.38	1.00	0.09	0.54	0.54	0.50	1.00	0.17
7	SRSF10	0.10	0.40	0.38	0.33	1.00	0.15	2.08	0.46	0.47	1.00	0.18
7	RP11-51J9.5	0.10	0.28	0.23	0.18	1.00	0.36	0.46	0.34	0.33	1.00	0.19
7	USMG5	0.27	1.43	0.44	0.40	1.00	0.13	1.77	0.64	0.61	1.00	0.24
7	PGLS	0.94	0.33	0.21	0.21	1.00	0.13	0.99	0.31	0.27	1.00	0.25
7	ZFP36L1	0.29	3.26	0.61	0.59	1.00	0.14	0.37	0.68	0.66	1.00	0.26
7	RPL31	0.15	1.26	0.96	0.94	1.00	0.42	10.00	0.98	0.98	1.00	0.27
7	ATP5D	0.16	1.14	0.56	0.52	1.00	0.15	2.40	0.69	0.66	1.00	0.29

7	HSPA8	0.16	2.44	0.81	0.83	1.00	0.39	42.72	0.90	0.89	1.00	0.29
7	ARGLU1	0.23	0.80	0.51	0.46	1.00	0.18	1.16	0.54	0.50	1.00	0.33
7	HIST1H1C	0.51	1.00	0.18	0.19	1.00	0.19	6.53	0.17	0.15	1.00	0.35
7	ANAPC16	0.20	0.62	0.38	0.39	1.00	0.44	0.29	0.58	0.57	1.00	0.37
7	SSR2	0.23	0.35	0.70	0.70	1.00	0.91	0.63	0.80	0.81	1.00	0.40
7	SNRPA	0.69	0.77	0.23	0.23	1.00	0.26	4.27	0.27	0.30	1.00	0.45
7	CYBA	0.38	9.93	0.84	0.84	1.00	0.28	6.53	0.91	0.91	1.00	0.48
7	UBL5	0.66	0.49	0.45	0.44	1.00	0.38	1.01	0.62	0.62	1.00	0.62
7	RPL27	0.39	0.33	0.95	0.94	1.00	0.69	22.03	0.98	0.98	1.00	0.63
7	DHCR7	0.42	0.38	0.16	0.14	1.00	0.48	0.52	0.13	0.13	1.00	0.66
7	COX5B	0.50	0.41	0.55	0.57	1.00	0.52	7.94	0.72	0.70	1.00	0.75
7	TSC22D3	0.69	1.07	0.91	0.91	1.00	0.51	2.26	0.88	0.87	1.00	0.76
8	TRGV10	0.00	2.00	0.25	0.15	1.00	0.00	2.00	0.07	0.14	0.70	0.00
8	FYB	0.22	0.26	0.33	0.31	1.00	0.01	2.45	0.50	0.47	1.00	0.03
8	NFKB2	0.01	0.39	0.26	0.19	1.00	0.31	1.56	0.25	0.22	1.00	0.03
8	EIF2S3	0.02	0.46	0.43	0.37	1.00	0.50	0.30	0.51	0.48	1.00	0.04
8	RNU12	0.02	2.01	0.40	0.30	1.00	0.90	0.72	0.26	0.25	1.00	0.05
8	CCNI	0.03	0.90	0.88	0.87	1.00	0.57	1.50	0.90	0.92	1.00	0.07
8	FXYD5	0.08	1.28	0.89	0.84	1.00	0.05	1.51	0.90	0.91	1.00	0.10
8	DYNLT1	0.07	0.43	0.22	0.19	1.00	0.84	0.25	0.39	0.38	1.00	0.14
8	SCGB3A1	0.11	0.25	0.11	0.10	1.00	0.16	0.25	0.14	0.11	1.00	0.21
8	PARK7	0.23	0.30	0.43	0.38	1.00	0.38	1.40	0.52	0.53	1.00	0.40
9	PPP1R14B	0.00	1.27	0.27	0.06	0.26	0.00	3.59	0.35	0.07	0.00	0.00
9	FAM177A1	0.00	1.47	0.71	0.45	0.04	0.00	2.96	0.82	0.36	0.00	0.00
9	GABARAPL1	0.01	0.67	0.52	0.31	1.00	0.00	1.84	0.65	0.25	0.00	0.00
9	YIPF5	0.21	0.29	0.25	0.17	1.00	0.00	1.24	0.46	0.17	0.00	0.00
9	CIB1	0.01	1.13	0.57	0.42	1.00	0.00	1.82	0.72	0.46	0.00	0.00

9	N4BP3	0.00	0.29	0.21	0.04	1.00	0.00	0.59	0.26	0.02	0.00	0.00
9	BIRC2	0.03	0.38	0.30	0.14	1.00	0.00	1.78	0.47	0.21	0.00	0.00
9	RGS2	0.11	0.82	0.39	0.26	1.00	0.00	9.36	0.47	0.33	0.00	0.00
9	PDCD4	0.00	1.21	0.75	0.55	1.00	0.00	2.87	0.82	0.62	0.00	0.00
9	EIF4EBP1	0.00	0.39	0.21	0.05	1.00	0.00	0.75	0.25	0.05	0.00	0.00
9	SLC7A5	0.00	2.07	0.68	0.34	0.02	0.00	1.25	0.44	0.22	0.00	0.00
9	OCIAD2	0.14	0.29	0.27	0.29	1.00	0.00	1.25	0.60	0.38	0.00	0.00
9	SYAP1	0.00	0.91	0.50	0.38	1.00	0.00	1.18	0.60	0.32	0.00	0.00
9	PPP1R16B	0.00	1.09	0.21	0.10	1.00	0.00	0.72	0.32	0.08	0.00	0.00
9	RBPJ	0.07	0.34	0.18	0.09	1.00	0.00	0.81	0.39	0.13	0.00	0.00
9	SKIL	0.02	0.39	0.25	0.11	1.00	0.00	1.07	0.43	0.17	0.00	0.00
9	CCSER2	0.00	0.58	0.25	0.27	1.00	0.00	1.69	0.46	0.27	0.00	0.00
9	RELB	0.00	0.83	0.55	0.33	1.00	0.00	1.67	0.60	0.37	0.00	0.00
9	FURIN	0.10	0.34	0.23	0.13	1.00	0.00	0.72	0.42	0.16	0.02	0.00
9	NFKB1	0.01	0.74	0.34	0.19	1.00	0.00	1.08	0.31	0.16	0.03	0.00
9	EML4	0.05	0.41	0.84	0.70	1.00	0.00	2.43	0.83	0.64	0.05	0.00
9	NDUFS5	0.02	0.80	0.57	0.48	1.00	0.00	1.71	0.71	0.56	0.10	0.00
9	ARID5A	0.00	0.94	0.55	0.38	1.00	0.00	0.74	0.63	0.39	0.18	0.00
9	PRKX	0.00	0.27	0.48	0.24	1.00	0.00	0.65	0.39	0.17	0.38	0.00
9	GCHFR	0.23	0.71	0.23	0.21	1.00	0.00	0.96	0.43	0.24	0.68	0.00
9	ABCG1	0.00	0.55	0.36	0.15	1.00	0.00	0.42	0.31	0.11	1.00	0.00
9	NINJ1	0.16	0.44	0.25	0.29	1.00	0.00	14.30	0.56	0.40	1.00	0.00
9	KDM6B	0.02	0.58	0.30	0.14	1.00	0.00	0.75	0.32	0.15	1.00	0.00
9	CD6	0.25	1.69	0.46	0.43	1.00	0.00	2.30	0.65	0.54	1.00	0.00
9	PTPN7	0.00	0.56	0.48	0.19	1.00	0.00	0.51	0.26	0.15	1.00	0.00
9	TNPO1	0.02	0.40	0.23	0.11	1.00	0.00	0.44	0.19	0.11	1.00	0.00
9	NCF1	0.01	0.66	0.30	0.13	1.00	0.00	0.81	0.25	0.17	1.00	0.00

9	REL	0.00	0.93	0.71	0.46	1.00	0.00	0.43	0.68	0.51	1.00	0.00
9	RBM8A	0.00	0.86	0.75	0.56	1.00	0.00	0.76	0.74	0.59	1.00	0.00
9	PLEKHA2	0.09	0.49	0.30	0.18	1.00	0.00	0.59	0.29	0.13	1.00	0.00
9	PSMA7	0.00	0.72	0.71	0.45	1.00	0.00	0.93	0.68	0.51	1.00	0.00
9	LEPROTL1	0.00	1.06	0.84	0.68	1.00	0.15	2.14	0.78	0.71	1.00	0.00
9	MTFP1	0.00	2.42	0.27	0.31	1.00	0.36	0.34	0.54	0.46	1.00	0.00
9	EPB41L4A-AS1	0.00	0.67	0.41	0.23	1.00	0.00	0.90	0.43	0.28	1.00	0.00
9	CXCR3	0.03	0.57	0.16	0.06	1.00	0.00	0.44	0.13	0.03	1.00	0.00
9	RASSF5	0.00	1.38	0.66	0.40	1.00	0.10	1.59	0.51	0.40	1.00	0.00
9	PDE4B	0.00	0.67	0.61	0.34	1.00	0.02	0.55	0.49	0.33	1.00	0.00
9	RAB8B	0.00	0.27	0.11	0.18	1.00	0.13	0.31	0.28	0.19	1.00	0.00
9	GNA15	0.00	0.58	0.21	0.05	1.00	0.00	0.60	0.11	0.05	1.00	0.00
9	CDC42SE2	0.25	0.52	0.50	0.45	1.00	0.00	0.80	0.64	0.49	1.00	0.00
9	FMNL1	0.23	0.28	0.46	0.36	1.00	0.00	0.42	0.61	0.42	1.00	0.00
9	DDX24	0.00	2.09	0.75	0.62	1.00	0.00	0.88	0.76	0.63	1.00	0.00
9	BCL7B	0.00	0.77	0.36	0.24	1.00	0.02	0.43	0.36	0.27	1.00	0.00
9	PRDX6	0.03	0.34	0.55	0.35	1.00	0.00	0.69	0.58	0.42	1.00	0.00
9	EMD	0.00	0.80	0.71	0.51	1.00	0.09	0.91	0.64	0.60	1.00	0.01
9	MAGOH	0.00	0.37	0.61	0.37	1.00	0.08	0.35	0.54	0.46	1.00	0.01
9	SQSTM1	0.00	2.66	0.73	0.58	1.00	0.00	7.17	0.75	0.64	1.00	0.01
9	DHCR7	0.00	1.14	0.25	0.14	1.00	0.02	1.32	0.17	0.13	1.00	0.01
9	STAT4	0.34	0.32	0.34	0.29	1.00	0.01	0.42	0.47	0.29	1.00	0.01
9	SERPINB9	0.01	0.43	0.30	0.12	1.00	0.10	0.26	0.18	0.12	1.00	0.01
9	ZDHHC3	0.07	0.26	0.16	0.12	1.00	0.01	0.33	0.25	0.12	1.00	0.01
9	CYLD	0.01	0.53	0.27	0.23	1.00	0.01	0.45	0.28	0.26	1.00	0.01
9	PPP2R2D	0.02	1.01	0.18	0.11	1.00	0.01	0.33	0.22	0.10	1.00	0.01

9	RNF115	0.01	1.54	0.48	0.28	1.00	0.01	0.59	0.42	0.27	1.00	0.01
9	ARRDC2	0.01	0.49	0.41	0.21	1.00	0.03	0.48	0.29	0.23	1.00	0.01
9	UPP1	0.01	0.64	0.25	0.21	1.00	0.01	0.48	0.32	0.19	1.00	0.01
9	RALGAPA1	0.22	0.28	0.27	0.18	1.00	0.01	0.48	0.24	0.14	1.00	0.01
9	PIK3IP1	0.01	1.37	0.68	0.53	1.00	0.02	0.97	0.68	0.53	1.00	0.02
9	YPEL5	0.01	0.67	0.75	0.56	1.00	0.06	1.26	0.56	0.46	1.00	0.02
9	U2SURP	0.01	0.61	0.36	0.26	1.00	0.21	0.84	0.32	0.34	1.00	0.02
9	TMEM259	0.01	0.64	0.39	0.21	1.00	0.06	0.31	0.29	0.21	1.00	0.02
9	MAFF	0.01	0.31	0.39	0.19	1.00	0.01	0.35	0.33	0.18	1.00	0.02
9	PNRC2	0.29	0.40	0.25	0.19	1.00	0.01	0.49	0.40	0.25	1.00	0.02
9	ARNTL	0.01	0.49	0.27	0.15	1.00	0.04	0.65	0.22	0.18	1.00	0.02
9	STRAP	0.09	0.49	0.46	0.39	1.00	0.02	0.28	0.61	0.44	1.00	0.03
9	SNRPA1	0.02	0.58	0.46	0.28	1.00	0.08	0.45	0.43	0.33	1.00	0.04
9	GYG1	0.13	0.30	0.46	0.31	1.00	0.02	0.53	0.50	0.35	1.00	0.05
9	EIF1B	0.02	0.89	0.43	0.27	1.00	0.06	0.62	0.44	0.37	1.00	0.05
9	CHD2	0.54	0.51	0.23	0.27	1.00	0.03	0.72	0.35	0.25	1.00	0.05
9	PET100	0.03	0.56	0.30	0.27	1.00	0.12	0.48	0.63	0.52	1.00	0.05
9	ETF1	0.08	0.41	0.39	0.24	1.00	0.03	0.40	0.22	0.21	1.00	0.06
9	EIF5	0.03	1.06	0.61	0.50	1.00	0.32	0.29	0.60	0.54	1.00	0.06
9	PLP2	0.04	1.47	0.41	0.25	1.00	0.03	0.64	0.43	0.30	1.00	0.06
9	ABI3	0.07	0.35	0.21	0.10	1.00	0.03	0.34	0.15	0.11	1.00	0.07
9	CHRAC1	0.04	0.26	0.30	0.15	1.00	0.18	0.32	0.25	0.17	1.00	0.07
9	APH1A	0.46	0.66	0.41	0.38	1.00	0.04	0.43	0.54	0.46	1.00	0.07
9	MECP2	0.08	0.30	0.18	0.14	1.00	0.04	0.48	0.33	0.23	1.00	0.08
9	ILF3	0.09	0.33	0.46	0.30	1.00	0.31	0.50	0.39	0.31	1.00	0.17
9	S100A11	0.21	0.29	0.32	0.22	1.00	0.09	0.27	0.40	0.29	1.00	0.17
9	SLC38A1	0.14	0.81	0.66	0.53	1.00	0.09	0.30	0.57	0.45	1.00	0.18

9	UBN1	0.10	0.88	0.21	0.17	1.00	0.61	0.50	0.17	0.17	1.00	0.19
9	P2RY8	0.11	0.45	0.50	0.37	1.00	0.16	1.34	0.53	0.44	1.00	0.20
9	PCGF5	0.16	0.32	0.34	0.24	1.00	0.12	0.31	0.32	0.26	1.00	0.22
9	ZNF292	0.14	0.45	0.25	0.19	1.00	0.23	0.57	0.28	0.23	1.00	0.26
9	EMC10	0.16	0.43	0.39	0.27	1.00	0.21	0.28	0.40	0.32	1.00	0.29
9	ZNF800	0.30	0.25	0.30	0.22	1.00	0.17	0.59	0.26	0.25	1.00	0.31
9	UBE2I	0.20	0.28	0.48	0.45	1.00	0.30	0.26	0.56	0.54	1.00	0.37
9	AMD1	0.21	0.29	0.34	0.25	1.00	0.49	0.96	0.38	0.35	1.00	0.37
9	KHDRBS1	0.96	0.32	0.43	0.45	1.00	0.36	0.36	0.42	0.46	1.00	0.59
9	ARIH2	0.48	0.28	0.36	0.30	1.00	0.38	0.47	0.40	0.34	1.00	0.62
9	SH2D1A	0.41	0.59	0.34	0.29	1.00	0.88	0.37	0.32	0.31	1.00	0.65
9	PPP1R2	0.86	0.41	0.34	0.35	1.00	0.60	0.91	0.38	0.32	1.00	0.84
9	GCC2	0.98	0.42	0.30	0.29	1.00	0.90	0.78	0.38	0.37	1.00	0.99

Summary table of genes identified to be differentially expressed between clusters identified by weighted nearest neighbours (WNN) analysis. Differential expression was performed using FindConservedMarkers in Seurat with batch as a grouping variable. Differential expression was performed using Wilcoxon Rank Sum test with Bonferroni correction for multiple testing (FDR = 0.01). P-values obtained from independently analysed batches were combined using Wilkinson’s method.
